# Can cash transfer interventions increase contraceptive use and reduce adolescent birth and pregnancy in low and middle income countries? A systematic review and meta-analysis

**DOI:** 10.1371/journal.pgph.0001631

**Published:** 2023-11-09

**Authors:** Dylan Kneale, Abel Kjaersgaard, Malica de Melo, Joelma Joaquim Picardo, Sally Griffin, Rebecca S. French, Helen E. D. Burchett

**Affiliations:** 1 EPPI-Centre, UCL Social Research Institute, University College London, London, United Kingdom; 2 International Centre for Reproductive Health Mozambique (ICRH-M), Maputo, Mozambique; 3 Department of Public Health, Environments and Society, Faculty of Public Health & Policy, London School of Hygiene & Tropical Medicine, London, United Kingdom; UFBA: Universidade Federal da Bahia, BRAZIL

## Abstract

Becoming pregnant and giving birth under the age of 20 is associated with a range of adverse social, socioeconomic and health outcomes for adolescent girls and their children in Low and middle income countries. Cash transfers are an example of a structural intervention that can change the local social and economic environment, and have been linked with positive health and social outcomes across several domains. As part of a wider review of structural adolescent contraception interventions, we conducted a systematic review on the impact of cash transfers on adolescent contraception and fertility. Fifteen studies were included in the review with eleven studies providing evidence for meta-analyses on contraception use, pregnancy and childbearing. The evidence suggests that cash transfer interventions are generally ineffective in raising levels of contraceptive use. However, cash transfer interventions did reduce levels of early pregnancy (OR 0.90, 95% CI 0.81 to 1.00). There was suggestive evidence that conditional, but not unconditional, cash transfers reduce levels of early childbearing. Given that much of the evidence is drawn from interventions providing cash transfers conditional on school attendance, supporting school attendance may enable adolescent girls and young women to make life choices that do not involve early pregnancy.

## Introduction

Becoming pregnant and giving birth under the age of 20 is associated with a range of adverse social, socioeconomic and health outcomes for adolescent girls and their children across a range of different settings, including elevated rates of maternal and neonatal mortality, with the negative health impacts in particular thought to be exacerbated in low income settings [1–3]. Reducing adolescent birth is a global priority, and an indicator of Sustainable Development Goal 3 (SDG3) to ‘ensure healthy lives and promote wellbeing for all at all ages’ [4]. Contraceptive use is a means of reducing levels of adolescent pregnancy through supporting greater reproductive choice.

Facilitating more extensive use of contraceptives to increase sexual and reproductive choice is dependent on ensuring a supply of contraceptives is in place as well as ensuring knowledge of and demand for contraceptives among adolescents through educational programmes [for example 5]. However, it is recognised that broader factors such as women’s empowerment and education can also be major ‘upstream’ determinants of use [6–8]. For example, preventing adolescent pregnancy also involves removing barriers to making informed reproductive choices including reducing gender inequality and discrimination, and creating health promoting environments through improving education and empowerment [8]. Many of these interventions may be structural in nature and involve altering the structural context—the social, economic, and political environments—through which health outcomes are produced and re-produced [9]. Cash transfers are an example of a structural intervention that have been used extensively in poverty alleviation campaigns to alter economic structures and improve access to income, as well as to increase school participation or reduce child marriage [10]. As part of a larger review looking at structural adolescent contraception interventions, in this paper we focus on cash transfers. We explore whether cash transfers provided to adolescent girls and/or their families result in higher levels of adolescent contraceptive use, or lower levels of early pregnancy or early childbearing.

### Cash transfer interventions and reproductive decision-making

Cash transfers have been linked with positive health and social outcomes across several domains including improving school attendance [11], decreasing levels of child labour [12]; childhood nutritional status [13, 14], and childhood health more broadly [15]; as well as a positive impact on mental health and subjective wellbeing [16]. Cash transfer interventions have also been linked to changes in reproductive decision-making. A previous review conducted by Khan and Hazra [17], which focussed on reproductive outcomes across women of different ages (as opposed to adolescent girls as is the case here), showed that cash transfer interventions had the potential to influence reproductive decision-making, with several studies included in their review indicating decreases in levels of pregnancy and increases in contraceptive use. Similar findings have also been noted in syntheses exploring these outcomes as part of a range of broader outcomes [18] and within reviews examining a suite of interventions [19]. However, Khan and Hazra [17] also found that there was substantial heterogeneity, even in the direction of the effect, precluding further quantitative synthesis. Their review also showed that much of the evidence base available evaluated the same (policy-level) intervention and consequently there was comparatively little evidence from smaller scale interventions and trials that may provide the basis for scaling up interventions to a policy or national level. Since then, the literature around cash transfer interventions has expanded (see [20]), justifying the need to synthesise the evidence further to explore their impact on reproductive health among adolescent recipients.

**Fig 1:**
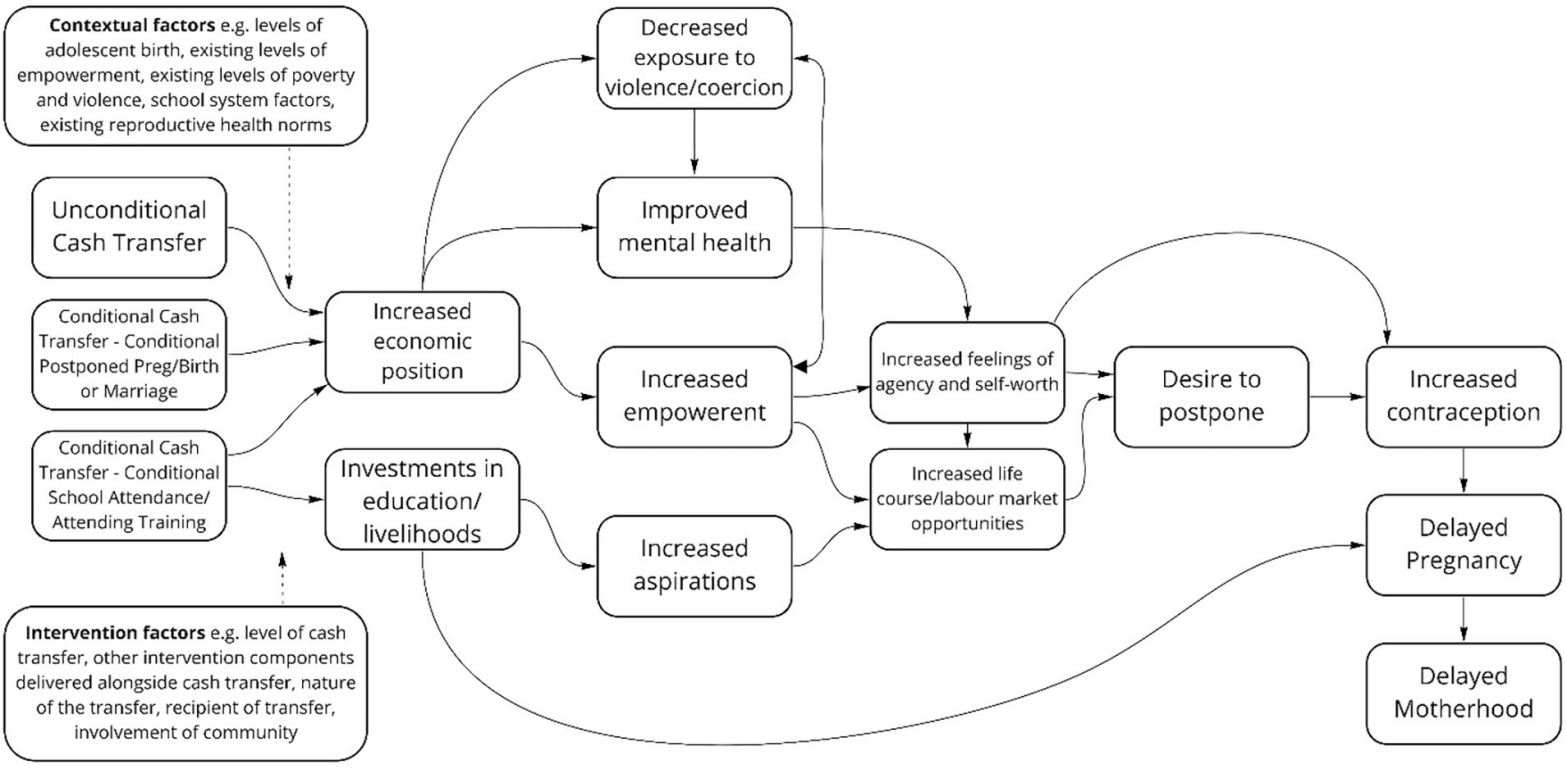
Logic model of CCT and UCT interventions.

A cash transfer is a broad term here reflecting three intervention models involving the transfer of economic capital to recipients: (i) conditional cash transfer; (ii) unconditional cash transfer; (iii) non-monetary transfer. A conditional cash transfer (CCT) reflects the situation where capital is transferred on the condition of fulfilling particular behaviours, e.g. attending school, or participating in intervention activities [17]. An unconditional cash transfer (UCT) is provided to recipients regardless of whether they fulfil any particular behaviour. We can also consider a further distinction where a CCT is provided to recipients on condition of fulfilling behaviour that is beyond the triallists direct control (e.g. postponing childbearing) compared to fulfilling behaviour that is linked to the triallists own activities (e.g. attending an intervention). We may also make a distinction between interventions that are focussed on the adolescent girl directly (e.g. through provision of direct payments to adolescent girls or that are conditional on changing girl’s behaviours) and those focussed on the households in which adolescent girls reside (e.g. where there are transfers to the head of household that improve the household’s economic status). Given that adolescent girls in some settings may not be in a direct position to receive such payments, and that direct payment options may fuel resentment or tension or risk the safety of adolescent girls [21, 22], other renumeration options may have been favoured by triallists and policy-makers with the intention of triggering similar mechanisms as is the case with monetary transfers (see Fig 1). To accommodate these situations, we expand our definition of cash transfers to include other forms of renumeration and non-monetary transfer–such as the provision of cooking oil that could support families–that have a transferable value beyond the adolescent girl herself. For example, while cooking oil is considered a commodity with broad currency that could be exchanged across a range of different households, so is considered a non-cash transfer, the payment of school fees or provision of school uniforms do not have the same broad transferable value within the local community. These non-monetary transfers could also be conditional or unconditional.

Cash transfers–a term we now use to reflect transfer of monetary and/or economic assets with transferrable value that are offered conditionally (CCT) or unconditionally (UCT)–are thought to impact adolescent reproductive outcomes through raising the financial status of the adolescent girl within the household and broader family, or encouraging particular behaviours, or raising the financial status of the household as a whole. We present a logic model of how cash transfers could impact reproductive outcomes in Fig 1 below, drawing on logic models presented within the cash transfer literature [23–25], but expanding on these to reflect different pathways through which CCT and UCT interventions could influence reproductive outcomes. Fig 1 shows one pathway hypothesising that increasing economic position activates positive changes in empowerment and a sense of agency and self-worth, which in turn increases life course and labour market opportunities which trigger a desire to postpone childbearing realised through increased contraception and delayed pregnancy. A second hypothesised pathway involves investments in education or in livelihoods which raises aspirations which also increase life course and labour market opportunities.

The extent to which the pathways shown in Fig 1 are activated by the intervention are theorised to be moderated by a set of (i) contextual factors (including, for example, adolescent fertility norms and gender norms) as well as (ii) intervention design factors (including, for example, the presence or absence of other intervention components that could comprise expanded contraceptive education and access, as well as the level and nature of the cash transfer). This study aims to explore whether cash transfer interventions are effective at increasing contraceptive use and reducing early pregnancy and birth.

## Objectives

The primary objective of this study is to assess the effectiveness of cash transfer interventions aimed at adolescent girls in increasing contraceptive use and reducing early pregnancy and early childbearing. In doing so, this review also (i) provides a description of the nature of these studies; (ii) estimates the size and direction of the quantitative impact of cash transfers in changing reproductive decision-making; and (iii) assesses the quality of the evidence base and identifies gaps and opportunities for further research in the field.

## Methods

### Protocol registration

The review is part of a larger review involving a mixed-method synthesis to develop a mid-range theory for structural adolescent contraception interventions, for which a protocol was registered on the International Prospective Register of Systematic Reviews (PROSPERO) (CRD42021254433).

### Ethics statement

Ethical approval was provided by the UCL Institute of Education Research Ethics Committee (REC 1442). The review involved synthesising published evidence in the public domain and therefore obtaining consent from trial participants was not applicable and not possible in this case.

### Search strategy and study eligibility

Studies included within the present review were screened from a larger systematic map of structural interventions to increase contraceptive use [20], which itself built on comprehensive searches and screening conducted for an earlier evidence gap map [26]. We describe the steps taken to create the systematic map in detail below, since this formed the source for the studies included in this review.

To create the updated map, we screened all the impact evaluations included in [26] related to contraceptive intervention or contraceptive outcomes. We then conducted a systematic search spanning from 2016 to July 2020 in eight databases, using controlled and free-text terms relating to adolescence, family planning, and low- and middle-income countries (LMICs); see S1 Appendix for full details of the search strategy. Due to language proficiency within the team, searches were limited to English or Portuguese language references. We limited the included papers in the updated map to those published in 2005 or later, since it was then that global interest in contraceptive use grew [27] as well as evaluations of structural sexual and reproductive health interventions [26]. We defined adolescence as spanning 10–19 years (and use the terms ‘adolescent girls’ and ‘adolescent girls and young women’ interchangeably to refer to refer to those aged 10–19) and used the World Bank’s definition to identify LMICs [28]. In addition, grey literature was sought from 16 websites (see S1 Appendix) and reference lists from relevant systematic reviews were screened.

The searches were conducted in June 2020 and we adopted a strategy of taking a broad sweep of different databases including with a heavy emphasis on searching and identifying sources of grey literature; this is contrast to strategies that might consist of a search of a narrower set of databases and a cursory exploration of the grey literature. This strategy paid dividends and within the broader review, approximately half of studies were identified as being grey literature (see [20]). However, it was not within the timescales and resources of the project to conduct updated searches and consequently to re-run the analyses and re-interpret the findings. Re-running updated searches and analyses after the project end data would have de-emphasised the collaborative nature of this work as a partnership between an NGO based in Mozambique and UK researchers, and we would not have benefitted from the insight of colleagues across the project team in identifying and interpreting evidence. While a less extensive targeted search of a narrow set of academic databases and a cursory exploration of grey literature may have supported an update within the project timescales, due to the nature of the literature in this field and where and how it is published, and the complexity of the intervention, this was not the strategy adopted here. Therefore, these results represent a snapshot of activity up to June 2020 only, and this is also highlighted as a potential limitation later in the study.

Search results were downloaded into Endnote and duplicates removed before being uploaded into EPPI-Reviewer [29] for screening. Each reference was screened for potential inclusion on the basis of title and abstract, using pre-specified exclusion criteria to ensure relevance. For the systematic map, records were excluded if they met any of the conditions below, with an additional condition added below for this review of cash transfer interventions.

- published before 2005
- reporting on studies not conducted in low- and middle-income countries, as defined by the World Bank in 2020
- not about reproductive health
- not evaluating a trial or policy
- not reporting on any of the following outcomes (abortion was not a target outcome in the map or the review, and even if it were included, would be underreported since abortion is illegal and/or highly stigmatised in many contexts):
  ○ uptake or use of modern contraception
  ○ intention/readiness to use contraception
  ○ desire to avoid, delay, space or limit childbearing,
  ○ desire to use contraception
  ○ pregnancy/birth
- not focused on adolescents; or did not contain at least half of participants within this age range or did not present stratified results
- not focused on structural interventions i.e. shaping girls’ economic or other empowerment, school enrolment and retention, shaping norms around gender, sexual behaviour or fertility, advocacy and other interventions to reduce gender and other inequalities
- not focussed on cash transfer interventions i.e. interventions involving the transfer of monetary and/or economic assets with transferrable value

To create the map, an initial sub-set of references were screened by four researchers (HB, SG, MM, JJP) to ensure consistency of understanding and application of criteria. Once at least 80% consistency had been achieved, the remaining references were screened individually. For those included at title/abstract screening stage, full reports were obtained and screened by two researchers (HB and either SG, MM, JJP or DK). Where agreement could not be reached, the paper was discussed with a third researcher from within the team. To identify cash transfer interventions, those coded as involving cash transfer interventions were rescreened by three reviewers (DK, HB and AK).

The map therefore included all interventions that were structural interventions to avoid or delay or prevent or limit or space the timing of pregnancy or childbearing or increase contraceptive uptake, including all cash transfer interventions. For this review, the studies within the map were screened by two reviewers to identify cash transfer interventions. This two-stage approach to identifying evidence where a map is used to initially characterise the evidence base, followed by the identification of smaller sub-sets of studies that can be used to answer focused questions through systematic reviews, is gaining traction across the evidence synthesis literature (for example see [30, 31]).

### Data extraction and quality assessment

After piloting, we extracted information from all included studies. Data on the name and nature of the intervention, the study design and period, the number and age of the participants, and the type of outcome measured were extracted initially (DK, HB, AK, MM, JJP, SG); outcome data were extracted by three reviewers (DK, HB, AK) who also conducted quality assessment first, before later calculating effect sizes. Each study was critically appraised using modified versions of two tools–the CASP tools for assessing RCT studies and for assessing cohort studies [32, 33]. Only parts of the tools that were focussed on the validity of the methods were used, and not parts reflecting on the results and their local applicability.

### Selecting and combining outcome data

Information on three primary outcomes, reflective of indicators of contraception and fertility outcomes, were extracted: (i) use of contraceptives (modern family planning methods); (ii) whether girls and young women had experienced a pregnancy; and (iii) whether girls had experienced a teenage birth. Data on post-test measures were prioritised for combining in meta-analysis and adjusted estimates of post-test measures were sought where available.

All studies meeting the criteria for study design and focus were included in this review. All data were extracted into EPPI-Reviewer 4, which was also used for calculating effect sizes including adjustments for clustering [34]. Stata was used for conducting further data transformations and robustness checks and for combining quantitative data [35]. Adjustments for clustering were made where this was not reported by trialists. Because no study included in the meta-analysis provided a direct estimate of the clustering effect through an intracluster correlation coefficient (ICC), an estimate of 0.05 was selected based on the ICC calculated in a recent survey of contraceptive practice in Malawi [36]. We expected outcomes to be reported using similar units of analysis, although in reality we encountered a number of variations and used Chinn’s formulae [37] for converting effect sizes and standard errors between standardized mean differences and odds ratios, following direction provided in the Cochrane Handbook [38]. For three studies, we adopted an approach analogous to creating a pseudo t-ratio through dividing a regression coefficient from a linear model by its standard error, and used this as the basis for creating an effect size. Although this is a possible solution to avoid losing data, this does have some limitations and can introduce ‘noise’, particularly with the inclusion of t-statistics from regression models that adjust for confounders compared to t-statistics from more descriptive group differences where there is no adjustment [39]. Our strategy is therefore to avoid losing data, although we examine the implications of this decision through sensitivity analysis. In the case of studies using Cluster Randomised Controlled Trial or Quasi-experimental designs, we prioritised extracting estimates that had been adjusted for baseline imbalances, although opted for unadjusted estimates where this was more appropriate (e.g. to account for shared control groups or where fewer transformations/assumptions would be made).

All data were initially combined using random-effects meta-analyses, as the underlying assumptions of a fixed-effects specification were not deemed to be compatible with the likely heterogeneity in intervention types and populations across studies. Where the between study heterogeneity was found to be negligible (zero), we denoted the model as a fixed effects model. While we prioritised data based on post-test measures where available, for a small number of studies only data on mean change (for example estimates from difference-in-difference models) were available and these estimates were meta-analysed separately.

We assessed statistical heterogeneity through examining the I^2^ measure and Cochran’s Q [38], and explored drivers of heterogeneity through conducting sensitivity and subgroup analyses. These subgroup analyses were based on study design, whether the study offered a conditional or unconditional cash transfer, and the broad geographical location of the study.

For publication bias, we plotted the distribution of studies’ effect sizes against their standard errors in a funnel plot for each outcome; we also undertook formal tests for small-study publication bias using Egger’s test [40]. However, these tests were likely underpowered for at least two of the outcomes (contraception and early childbearing). Further sensitivity analyses were undertaken on the basis of using fixed-effects compared to random effects modelling, and the impact of studies using a randomised design versus quasi experimental.

## Results

### Study selection

**Fig 2:**
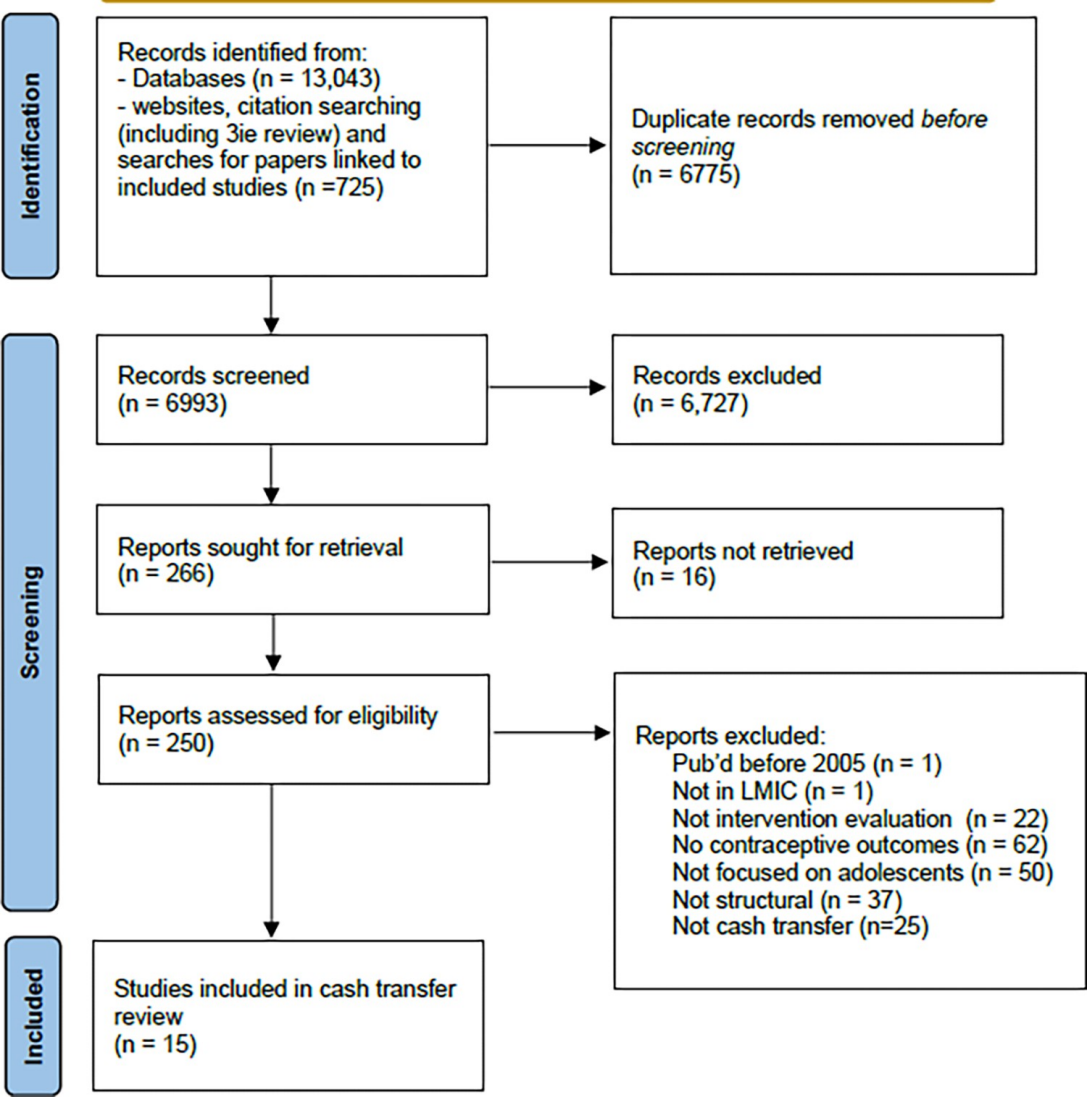
Flow of studies through the review.

The search was conducted in 2020 and after de-duplication, the titles and abstracts of 6,993 outcome evaluation studies were screened with 250 full-text records assessed for eligibility as potential structural interventions, and fifteen outcome evaluation studies eligible for inclusion (Fig 2).

Some studies were deemed to be close to meeting the inclusion criteria but were excluded as they did not meet our definition of a cash transfer. This included where the study had no relevant outcomes [41]; where the transfer was in the form of a loan [42, 43] or where the transfer was in the form of schools fees or scholarships which were not transferrable (e.g. within the broader household) or of low monetary value [44–46] or in the form of sexual health vouchers that were not broadly transferrable [47]. Two other linked studies were excluded as they evaluated the timing of a receipt of cash transfer rather than the impact of receipt (versus not receipt) [48, 49].

### Characteristics of studies and risk of bias

Fifteen studies were identified (Fig 1). Three of these studies included reports of interventions conducted in different settings: Stecklov, Winters [50] reported on interventions conducted in three different countries and Dake, Natali [23] reported on studies conducted in two different countries; meanwhile Austrian, Soler-Hampejsek [25] reported on interventions conducted in two different settings in Kenya although using different evaluation methods (an RCT in one setting and a cluster RCT in another). Due to these substantial differences in country and evaluation methods, we view these as being substantial enough to constitute different ‘studies’ (not just different arms) in our description of studies below. Therefore our description below is based on 19 ‘studies’.

#### Characteristics of studies

The majority of studies were experimental, with 11 of 19 studies or study arms constituting cluster randomised trials and two studies randomising by individual adolescent; a further six studies adopted a quasi-experimental (non-randomised) or natural experiment design. Most studies had taken place in sub-Saharan Africa (Kenya (3), Malawi (3), Ethiopia (1 study), Liberia (1), Zambia (1) and Zimbabwe (1)); with a smaller number of studies conducted in Asia (Bangladesh (1), Pakistan (1)), and South or Latin America (Mexico (2), Nicaragua (2), Brazil (1), and Honduras (1),).

The majority of studies evaluated CCT interventions (15/19 studies), with only four studies considering UCT interventions; including one study that considered both UCT and CCT interventions in separate arms [51]. The majority of CCT interventions were fully or partially conditional on behaviours or interventions not directly within the triallists control (12 of 19 studies in total); in nine of these studies, the transfer was conditional on enrolment or attendance at school; receipt of the cash transfer was conditional on postponement of marriage in two studies (along with attendance at support groups to encourage girls to stay in school in one study [52]); while in another four studies the transfer was conditional on both attendance and health service utilisation requirements. Clearly, in the majority of interventions, remaining in school was viewed as a key mechanism to adopting contraception and postponing pregnancy or childbearing. In five of the studies, receipt of the cash transfer was conditional on participating in intervention components, rather than external conditions such as schooling or marriage.

As would be expected, most studies involved the transfer of money directly into the hands of adolescent girls or their families (n = 15), with some specifying that the transfer had to occur to particular family members [24] or to female members of households [53]. In contrast, for three studies the transfer consisted of transferrable assets of worth to the household (for example a substantial quantity of cooking oil [54]) while a further study involved the transfer of small amounts of cash in addition to the transfer of a goat to the household [55].

In just over half of studies (12/19), the cash transfer was available along with other support or interventions provided by triallists (including where the transfer was conditional on attendance in another part of the intervention). Within this number we also include four studies (with one of the studies including evaluations conducted in three different countries) where the conditional cash transfer aimed at keeping adolescent girls in school was offered alongside an additional cash transfer that was conditional on attending health information sessions and/or ensuring that children aged under 5 received health checks [50, 53]. In other cases, the cash transfer was offered alongside other intervention components that addressed the social determinants of health including violence prevention [25], health education [25, 55–57], empowerment [21], school-focussed support groups [52], and broader life and employment skills [25, 55, 57, 58]. Not only was there heterogeneity in the intervention, there was also heterogeneity in the comparison, e.g. some compared the cash transfer to an active control, others to no intervention, as well as clear differences in contexts) (see Table 1). While such differences in the form of interventions may be typical in these forms of complex, upstream, social interventions, they nevertheless underscore the inherent challenges in understanding the impact of this suite of interventions.

**Table 1:** Study characteristics–for more detailed description see S2 Appendix.

Study authors	Setting	Design	Treatment arms	Condition	Additional intervention	Cash or transferrable asset	Control	Data used in meta-analysis
Alam et al (2011) [54] Punjab Female School Stipend Program	Pakistan	Quasi-experimental	One	Conditional on school attendance	None	Cash	N/A (pre-post)	No
Austrian et al (2020) [25] Adolescent Girls Initiative–Kenya	Kenya (Urban-Kibera)	RCT	Three	Conditional on school attendance	Yes—dependent on study arm. All three involved violence prevention and education; one involved additional health intervention and a second involved an additional health intervention and wealth creation programme	Cash	Active control—violence prevention involving communities identifying strategies to reduce violence towards women and girls	Yes
Austrian et al (2020) [25] Adolescent Girls Initiative–Kenya	Kenya (Rural-Wajir)	CRCT	Three	Conditional on school attendance	Yes—dependent on study arm. All three involved violence prevention and education; one involved additional health intervention and a second involved an additional health intervention and wealth creation programme	Cash	Active control—violence prevention involving communities identifying strategies to reduce violence towards women and girls	Yes
Baird et al (2012) [51] Zomba Cash Transfer Program	Malawi	CRCT	Two: An unconditional cash transfer arm and a conditional cash transfer arm	Conditional arm: Conditional on school attendance (trial included an unconditional arm also)	None	Cash	Inactive control (control received nothing)	Yes
Barham et al (2018) [59] Red de Protección Social (RPS)	Nicaragua	CRCT	One	Conditional on school attendance	Yes—additional (larger) payments to support nutrition and health conditional on health checks for children under 5 and attendance at health information sessions of heads of household	Cash	Waitlist control	No
Buchman et al (2016) [21] Jibon-O-Jibika Program (with Kishoree Kontha (KK), or “Adolescent Girl’s Voice”)	Bangladesh	CRCT	Two: One arm offered an additional empowerment intervention	Conditional on postponement of marriage	Yes—dependent on study arm	Transferrable high worth asset (cooking oil)	Inactive control (control received nothing)	Yes
Dake et al (2018) [23] Social Cash Transfer Program (SCTP)	Malawi	CRCT	One	Unconditional	None	Cash	Inactive control (control received nothing)	Yes
Dake et al (2018) [23] Multiple Categorical Targeted Grant (MCTG)	Zambia	CRCT	One	Unconditional	None	Cash	Inactive control (control received nothing)	Yes
Darney et al (2013) [53] Oportunidades—established by the Mexican government in 1997 as PROGRESA	Mexico	Quasi-experimental	One	Conditional on school attendance and family visits to health centres	None	Cash	N/A (pre-post)	Yes
Dunbar et al (2014) [57] Shaping the Health of Adolescents in Zimbabwe (SHAZ!)	Zimbabwe	RCT	One	Conditional on completion of vocational training programme and development of business plan	Yes—a combined intervention package including life-skills and health education, vocational training, micro-grants (conditional cash transfer) and social supports	Transferrable high worth asset (capital equipment, supplies or training)	Active control—life-skills and health education	Yes
Erulkar (2009) [52] Berhane Hewan	Ethiopia	Quasi-experimental	One	Conditional on postponement of marriage and attendance of girls at meetings	Yes—support groups to encourage girls to stay in school	Cash and Transferrable high worth asset (a goat)	Inactive control (Matched control received nothing)	Yes
Handa et al (2015) [24] Kenya Cash Transfer for Orphans and Vulnerable Children	Kenya	CRCT	One	Unconditional	None	Cash	Waitlist control	Yes
Martinez-Restrepo (2012) [60] Young Agent Project in Brazil (Projeto Agente Jovem)	Brazil	Quasi-experimental	One	Conditional on school attendance	None	Cash	Inactive control (Matched control received nothing)	Yes
Mercycorps (2015) [55] Sawki programme	Niger	Quasi-experimental	Two	Conditional on girls attending training and meetings	Yes—dependent on study arm. Both arms offered additional health education (nutrition and reproductive health) for girls and one offered additional life skills education	Transferrable high worth asset (50kg bag of lentils)	Inactive control (Matched control received nothing)	No
Özler et al (2020) [58] Girl Empower	Liberia	CRCT	Two	Conditional on girls attending training and meetings	Yes—Both arms offered additional life skills education; one arm offered additional payments for caregivers of adolescent girls	Cash	Inactive control (Matched control received nothing)	Yes
Rosenberg et al (2018) [56] Girl Power Malawi	Malawi	Quasi-experimental	One	Conditional on girls attending training and meetings	Yes—girls were offered access to a youth focussed health service and educational sessions	Cash	Inactive control (control received treatment as usual)	Yes
Stecklov et al (2006) [50] Education, Health and Nutrition Program- Progresa	Mexico	CRCT	One	Conditional on school attendance and family visits to health centres	Yes—additional payments to support nutrition and health conditional on health checks for children under 5 and attendance at health information sessions of heads of household	Cash	Not described	No
Stecklov et al (2006) [50] Family Assistance Program (PRAF)	Honduras	CRCT	One	Conditional on school attendance and family visits to health centres	Yes—additional payments to support nutrition and health conditional on health checks for children under 5 and attendance at health information sessions of heads of household	Cash	Not described	No
Stecklov et al (2006) [50] Social Protection Network (RPS)	Nicaragua	CRCT	One	Conditional on school attendance and family visits to health centres	Yes—additional payments to support nutrition and health conditional on health checks for children under 5 and attendance at health information sessions of heads of household	Cash	Not described	No

See S2 Appendix for further characteristics of studies.

#### Risk of bias

Study quality varied and most frequently the review team identified concerns among some studies around baseline equivalence (among cluster RCTs). Some studies using quasi-experimental designs, although in many ways well-conducted, were deemed to be less likely to accurately estimate intervention effects as they relied on cross-sectional data with matched designs. Most studies did not provide much detail on the measurement tools used to collect information on contraceptive, pregnancy and birth outcomes, and therefore we are unable to comment on the reliability of these instruments. In addition, studies were not always clear on whether outcomes around contraceptive use and fertility were collected from (currently) sexually active young women or not. There is a risk that some of the impacts observed between intervention groups may be confounded by differences in the frequency of sexual activity, particularly as some of the studies may have also increased levels of abstinence (which was not directly under consideration here). We were unable to disentangle the extent to which this form of confounding influenced the results and could not produce a refined estimate of the extent to which studies influenced contraceptive practice among those who were sexually active.

Finally, while nineteen ‘studies’ were identified as eligible, data from only fourteen studies were able to be extracted for synthesis. Data from interventions in Honduras and Nicaragua were unable to be included in quantitative synthesis either because we were unable to assess the impact of exposure, or where the data did not provide an estimate for the age group of interest [50, 59]. For a further study, MercyCorps [55], we only describe the results for contraceptive use narratively as we were not able to incorporate the results from two arms in the main meta-analytic model (the results explicitly assessed change in usage rather than post-test assessment); we were not able to incorporate the results from Alam, Baez [54] in the main meta-analytic model examining early childbearing for the same reason. Conversely, a number of studies contributed information about different arms of an intervention, with studies by Baird, Garfein [51], Austrian, Soler-Hampejsek [25], and Buchmann, Field [21] providing multiple effects sizes on different intervention arms or different population groups in meta-analytic models (note that where arms were comparing to a common control, adjustments were made to the effective sample size in line with meta-analytic practice).

### Estimates of impact–contraceptive use

**Fig 3:**
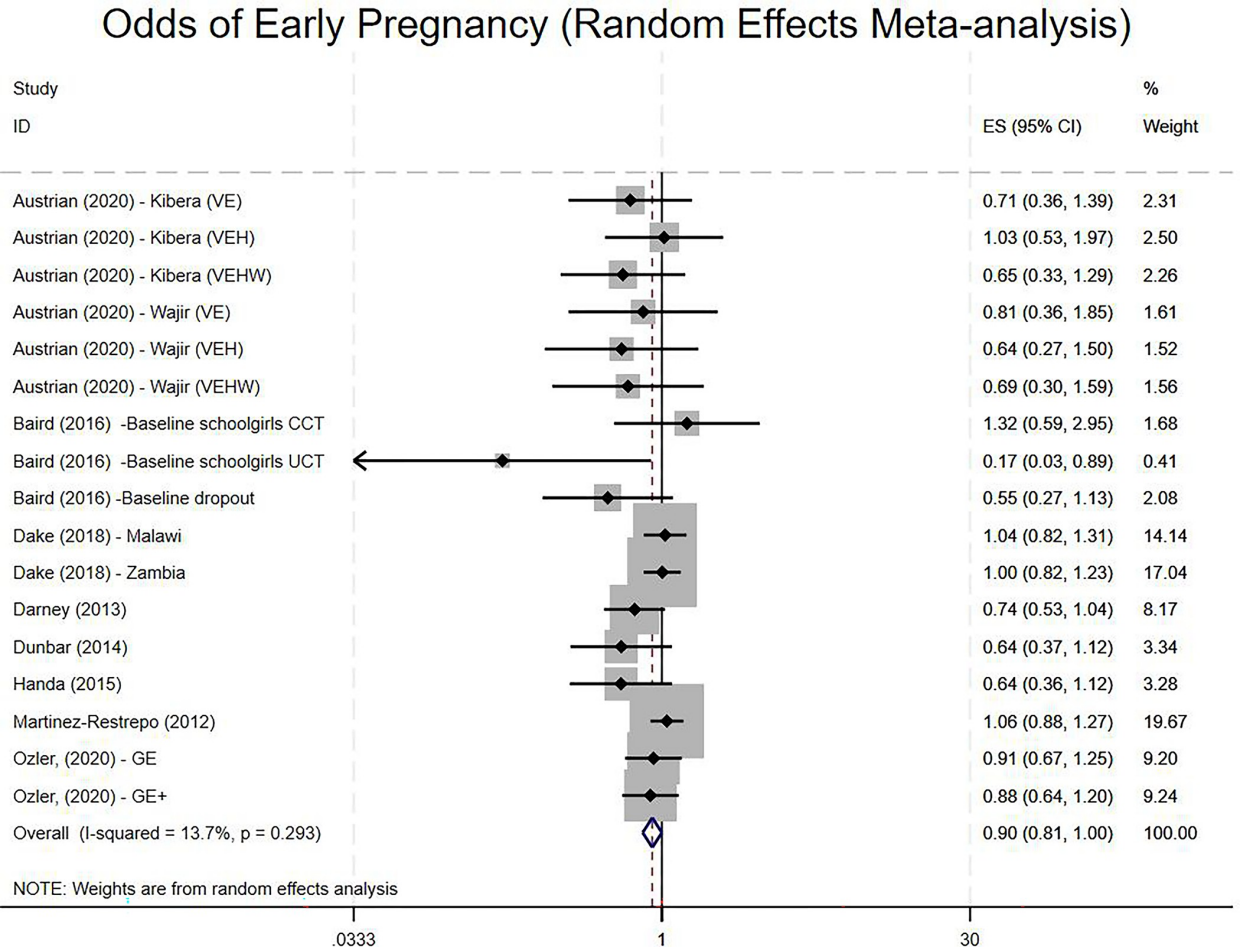
Random effect model of the odds of contraceptive use at post-test. **Key**: VE: Violence Prevention and Education (involving cash transfer); VEH: Violence Prevention and Education (involving cash transfer) and Health Intervention; VEHW: Violence Prevention and Education (involving cash transfer) and Health Intervention and Wealth Creation [Kibera and Wajir refer to two sites in Kenya].

Eleven studies and arms provided enough information on contraception to calculate an effect size for meta-analysis, looking at the odds of using contraceptive methods at post-test (Fig 3). This showed that a number of interventions indicated a positive effect, with one study in particular [56], suggestive of a large and positive impact of the intervention on contraceptive use. Overall, however, while the evidence was tentatively suggestive of a positive impact of cash transfers on an increase in contraceptive use, the results were ultimately inconclusive (OR 1.40, 95% CI 0.79 to 2.48; Fig 1); a fixed effect model also suggested a positive impact although was deemed inappropriate given the high levels of heterogeneity. This level of this heterogeneity (I^2^ (82.0%) allowed for further exploration of the potential drivers of this heterogeneity. Sub-group analyses based on study design characteristics, the type of cash transfer (conditional vs unconditional; there was no variation in this model); the type of conditionality (external to the trial or linked to triallists’ activities); and the geographic region did not help to explain heterogeneity. Additional subgroup analyses (based on reviewer feedback) examining whether the cash transfer was part of a larger intervention and whether the control arm was active or inactive were also uninformative in explaining heterogeneity. Although based on a relatively small number of studies, neither the funnel plot nor Egger’s test were indicative of publication bias. Further sensitivity analyses explored the impact of removing or trimming the value of Rosenberg on the analysis and particularly the level of heterogeneity, although I^2^ values remained consistently high after doing so. Similarly, sensitivity analyses exploring the impact of removing effect sizes that had undergone Chinn’s transformation or those that were based on the pseudo-t approach–in this case just Martínez-Restrepo [60]–did not reveal any substantial change in the direction or magnitude of the effect size or on heterogeneity statistics.

Finally, while not combined in meta-analyses, data reported by [55] suggested that two arms of an intervention that involved cash transfers led to a decrease in the use of contraceptives based on difference-in-difference analyses, although neither effect was statistically significant.

Our results therefore suggest that the impacts of cash transfers on contraceptive use are heterogeneous, and while suggestive of a positive impact overall, are ultimately inconclusive. Furthermore, cash transfers appear to *lower* the odds of contraceptive use in some settings, although the extent to which this reflects measurement error, contextual effects, or intervention design is unclear, although in the case of the study by Darney, Weaver [53] this is likely due to the level of sexual activity (see [6]).

### Estimates of impact–pregnancy

Information from seventeen study arms contributed to a meta-analysis of pregnancy outcomes. Pregnancy experiences were measured differently across the studies; while an ideal measure might reflect whether sexually active girls had experienced a pregnancy since the intervention, we instead extracted data on whether intervention recipients had ever experienced a pregnancy (eleven estimates from five studies [23–25, 53, 58]); whether intervention recipients had experienced pregnancy as a teenager [60]; whether adolescent girls had experienced an unintended pregnancy [57], and whether the adolescent girls were currently pregnant at the time of follow up (two estimates from one study [61]).

**Fig 4:**
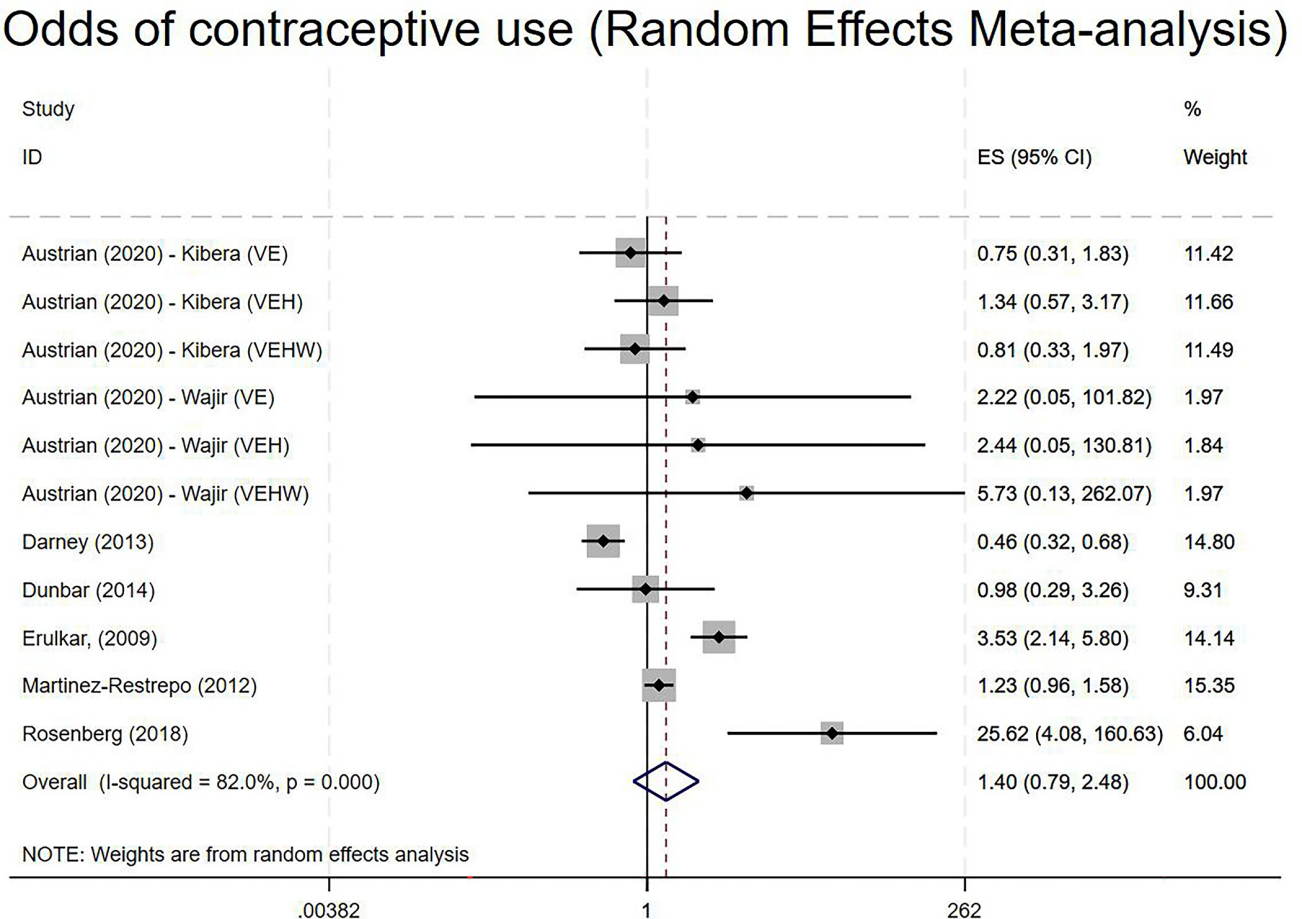
Random effect model of the odds of having experienced pregnancy at post-test. **Key**: VE: Violence Prevention and Education (involving cash transfer); VEH: Violence Prevention and Education (involving cash transfer) and Health Intervention; VEHW: Violence Prevention and Education (involving cash transfer) and Health Intervention and Wealth Creation [Kibera and Wajir refer to two sites in Kenya]; Baseline schoolgirls = in school at start of intervention; Baseline dropouts = dropped out of school at start of intervention; CCT = Conditional Cash Transfer; UCT = Unconditional Cash Transfer; GE: Girl Empower (involving cash transfer); GE+ = Girl Empower Plus (involving additional cash transfer).

Overall, the evidence suggested that receipt of a cash transfer intervention reduced the odds of experiencing an early pregnancy relative to the control group (OR 0.90, 95% CI 0.81 to 1.00; Fig 4) with low heterogeneity (I^2^ = 13.7%). Subgroup analyses based on study design and type of cash transfer, including whether the cash transfer was part of a larger intervention and whether the control arm was active or inactive, suggested that these factors did not help to explain between study heterogeneity. Strikingly, a large group of Conditional Cash Transfer studies suggested that there was very little heterogeneity in impact (13 effect sizes; OR 0.90, 95% CI 0.80 to 1.00; I^2^ = 0%); although a small group of studies evaluating Unconditional Cash Transfers suggested large amounts of heterogeneity and uncertain effectiveness (4 effect sizes; OR 0.90, 95% CI 0.68 to 1.19; I^2^ = 56.0%).

Further sensitivity analyses exploring the impact of removing effect sizes that had undergone Chinn’s transformation or those that were based on the pseudo-t approach–in this case Dake, Natali [23], Martínez-Restrepo [60], Özler, Hallman [58]–did not reveal any substantial change in the direction or magnitude of the effect size or on between study heterogeneity, which remained very low (OR 0.88, 95% CI 0.78 to 0.98). A funnel plot and tests for publication bias suggested that there was evidence of small-study effects, and presents a potential caveat to these results. However, it is also worth noting that nine of the seventeen data points originated from trials reported in grey literature (working papers and reports), including one thesis [60], which could help to mitigate concerns about small study impacts.

### Estimates of impact–early childbearing

**Fig 5:**
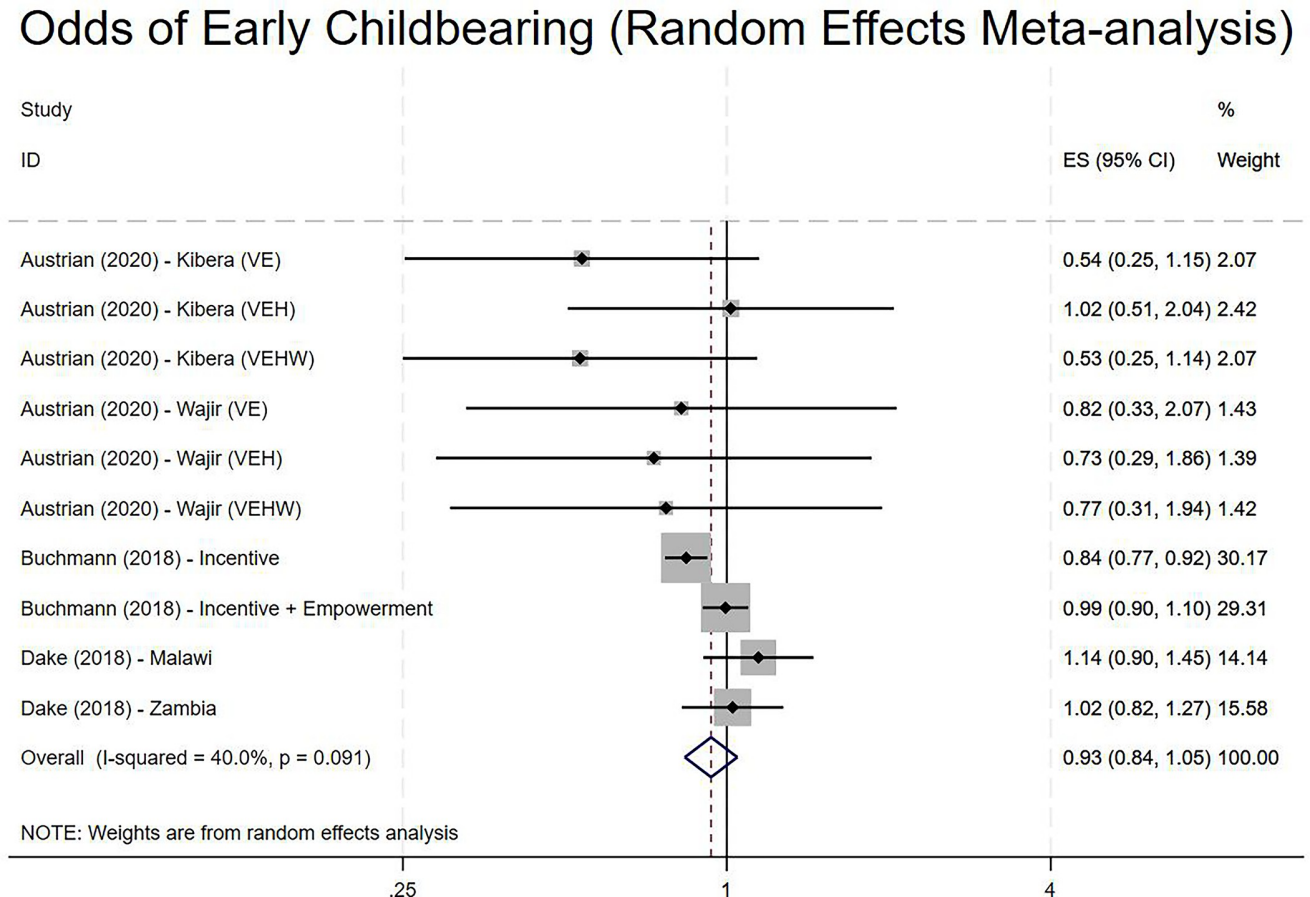
Random effects model of the odds of reducing early childbearing. **Key**: VE: Violence Prevention and Education (involving cash transfer); VEH: Violence Prevention and Education (involving cash transfer) and Health Intervention; VEHW: Violence Prevention and Education (involving cash transfer) and Health Intervention and Wealth Creation [Kibera and Wajir refer to two sites in Kenya].

Finally, we examined the impact of cash transfer interventions in reducing the odds of early childbearing. Here we drew on ten data arms originating from three studies. Overall, the analysis found that cash transfer interventions were not effective in reducing the odds of early childbearing (OR 0.93, 95% CI 0.84 to 1.05; Fig 5), albeit with moderate heterogeneity. Subgroup analysis based on whether the cash transfer was conditional or unconditional provided suggestive, although ultimately inconclusive, evidence that CCTs were more effective in reducing the odds of early childbearing (OR 0.88, 95% CI 0.78 to 1.01; I^2^: 32.3%) than UCT interventions (OR 1.08, 95% CI 0.92 to 1.27; I^2^: 0%). Other subgroup analyses, including whether the cash transfer was part of a larger intervention and whether the control arm was active or inactive, were uninformative in explaining between study heterogeneity.

As was the case for the earlier analyses, a funnel plot and tests for publication bias suggested that there was evidence of small-study effects, and presents a potential caveat to these results. In this analysis several of the arms originated from trials reported in grey literature (working papers and reports).

The results from Alam, Baez [54], which examined mean change in the probability of giving birth through difference-in-difference could not be included in the meta-analysis. However their findings suggest that the intervention had no impact of the probability of having given birth between baseline and follow-up.

Overall the evidence is suggestive that CCT could lead to lower incidence of early childbearing but not UCT, albeit with some caveats and the overall impact of cash transfers on early childbearing remains uncertain.

## Discussion

### Summary

This review summarises the effectiveness of cash transfer interventions focussed on adolescent girls and young women in increasing contraceptive use and reducing early pregnancy and childbearing. Through its focus on adolescent girls and young women, and the focus on publications after 2005, this review has a narrower remit than other recent systematic reviews in this area [17, 62]. Despite this, the results here represent the largest body of synthesised research on the impact of cash transfers (15 studies were identified in total) on contraceptive and fertility outcomes, and one of the few reviews to provide a quantitative estimate of the impact (with the largest meta-analysis including data from 15 studies and 17 study arms).

With respect to our first objective in understanding the characteristics of the literature in this area, most of the studies have taken place within sub-Saharan Africa. However, only one of those studies focussed on a country in the top 5 globally with the highest levels of adolescent births consistently over the past decade (MercyCorps [55] in Niger [63]). We find that most studies conducted in this area have focussed on the effectiveness of *conditional* cash transfers, as opposed to *unconditional* cash transfers. Among these conditional cash transfers, most transfers have taken place conditional on girls enrolling in and attending school.

Evidence in this review suggests that providing cash transfers can reduce early pregnancy. Previous reviews have highlighted that cash transfer interventions are effective in improving school outcomes, with tentative evidence to suggest that *conditional* cash transfers are particularly effective in improving educational outcomes [11]. The evidence in this review is also broadly supportive that conditional transfers are more effective, with subgroup analyses suggestive that a review focussed solely on conditional cash transfers only would identify lower levels of pregnancy (with no between study heterogeneity) and lower levels of early childbearing. This suggests that the improvements in school attendance (not directly considered here) that can follow conditional transfers may also extend to changing life course directions more broadly, through enabling adolescent girls and young women to make choices that do not involve early pregnancy (see Fig 1). This could be through staying on longer in school, and so delaying marriage and sexual activity, and later through increased participation in the labour market, which may change preferences for early childbearing as well as their agency to control their sexual and reproductive choices. Further work is needed to understand the mechanisms through which cash transfers may exert an impact, and in particular how cash transfer interventions should be designed to correspond with appropriate life stages of recipients, and which contextual conditions best support cash transfers as an appropriate intervention to lower the age at first birth, and the extent to which [6]. While we could therefore hypothesise that cash transfers are an example of an effective upstream intervention, other features of the review need to be considered and investigated before recommendations can be made around these findings; these represent some of the limitations of the review discussed below.

Firstly, we observe little evidence that cash transfer interventions improve the uptake of contraception, although our results also suggest that the greatest intervention impacts are on pregnancy (and birth in the case of CCT). Although many studies explore contraceptive use as a (main) determinant of pregnancy and birth, structural interventions also influence the frequency of (vaginal) sexual activity [6], and cash transfer interventions may also influence the latter more than the former. A second limitation is around how much of the impact we can attribute to the cash transfer itself, given that in half of the studies, the transfer was provided alongside other intervention activities. More broadly, there was heterogeneity across the interventions across several dimensions including study design, control conditions, intervention type, and context. The relatively small number of studies preclude in-depth exploration of the implications of these differences. Similarly a third limitation is that we are unable to fully distinguish the impacts of *unconditional* from *conditional* cash transfers due to the low numbers of the former type of transfer in the review. For pregnancy as an outcome, there were tentative indications that the impact of UCTs were more ambiguous and heterogeneous (see Dake, Natali [23], Baird, Garfein [51] and Handa, Peterman [24] in Fig 4 and description of subgroup analysis); similarly for early childbearing there was tentative evidence that CCTs may be effective in reducing early birth but not UCTs. Nevertheless, further work is needed to elucidate the mechanisms and to establish if these mechanisms differ between unconditional and conditional transfers. For example, studies drawing on UCTs were observed to focus on very vulnerable households, although this was not a feature in some of the descriptions of CCT interventions, and further work is needed to assess if UCT interventions might be more appropriate and effective for some households compared to CCT interventions.

A fourth limitation of the results here surrounds possible publication bias for pregnancy and birth models, and the potential that the findings to over-represent studies with a positive impact. However, the extensive searching and screening, and the high representation of data from grey sources does help to limit the possibility of publication bias. The extensive searches of grey literature did constrain the ability of the team to update the searches after June 2020. In addition, no publication bias was detected for models of contraception, despite having the same approach to searching and selecting data and despite substantial overlap between the studies in the models. A final limitation is around the possibility that the focus on cash transfers here could be viewed as a deviation from the protocol for the main review which sought to examine all upstream interventions. However, the evidence in this sub-study synthesises evidence from conceptually similar studies suitable for meta-analysis in order to contribute to the broader theory development of the main study. In this respect, focussing only on those studies viewed as being similar enough for meta-analysis is no different from common meta-analytic practice. Where the analysis plan did deviate was in the conduct of subgroup analyses that were not pre-specified and in this respect, these do constitute a deviation.

### Conclusions

Currently, over 140 women per 1000 give birth between the ages of 15–19 years in 22 countries (for reference this number is 12 per 1000 in the UK) [63]. This review suggests that offering cash transfers, primarily to encourage school attendance, may be an effective strategy in reducing the odds of early pregnancy, although the evidence in terms of increasing contraceptive use is not conclusive. Although the reduction in the odds of early pregnancy may be viewed as relatively modest, this may nevertheless have substantial impacts on a population level. Cash transfers are likely to be particularly effective where social systems support educational and labour market opportunities for young women. Further work is needed to understand the mechanisms through which cash transfers exert an influence, and which types of support may be needed alongside cash transfers to ensure impacts are observed.

## Supporting information

S1 Checklist PRISMA checklist. (DOCX)

S1 Appendix Search strategy. (DOCX)

S2 Appendix Characteristics of studies & quality assessment tables–CASP tool for cohort studies, quality assessment tables–CASP tool for RCT studies. (DOCX)
