# Supplementary material for: Can cash transfer interventions increase contraceptive use and reduce adolescent birth and pregnancy in low and middle income countries? A systematic review and meta-analysis
Source: PLOS Glob Public Health. 2023 Nov 9;3(11):e0001631. doi: 10.1371/journal.pgph.0001631 (PMC10635429; doi:10.1371/journal.pgph.0001631)
Supplement: S1 Appendix — (DOCX) [file pgph.0001631.s002.docx]

# Appendix 1: Search strategy

**Databases searched**

The following bibliographic databases were searched on 29 & 30 July 2020.

- OvidSP Medline ALL, 1946 to July 27, 2020.
- OvidSP Embase, 1947 to 2020 July 29.
- OvidSP Global Health, 1910 to 2020 week 29.
- Ebsco CINAHL Plus, complete database to search date.
- Ebsco Africa-Wide Information, complete database to search date.
- Clarivate Analytics Web of Science, Science Citation Index-Expanded. 1970-present, data last updated 2020-09-16.
- ProQuest ERIC, 1966-search date.
- WHO Global Index Medicus, complete database to search date.

**Websites hand searched**

| 1. Advocates for Youth |
| --- |
| 1. Family Health International |
| 1. Guttmacher Institute |
| 1. Interagency Youth Working Group |
| 1. International Center for Research on Women |
| 1. International Planned Parenthood Federation |
| 1. Family planning high impact practices |
| 1. Marie Stopes International |
| 1. Pathfinder International |
| 1. Population Council, |
| 1. United Nations Population Fund |
| 1. United Nations Children’s Fund |
| 1. World Health Organization (WHO) |
| 1. NBER |
| 1. World Bank (2016 onwards) |
| 1. JSI (2016 onwards) |

**Example search strategy: Medline OvidS**P

1. adolescent/ or child/ (2806512)
2. puberty/ or menarche/ (17517)
3. homeless youth/ (1290)
4. minors/ (2576)
5. disabled children/ (6288)
6. students/ (58686)
7. child*.ti,ab. (1383127)
8. (girl or girls or boy or boys).ti,ab. (229162)
9. (paediatric* or pediatric*).ti,ab. (350866)
10. (schoolage* or (school adj1 age*)).ti,ab. (22762)
11. minor*.ti,ab. (295741)
12. ((school or college) adj3 (pupil* or student*)).ti,ab. (46075)
13. prepubescen*.ti,ab. (1008)
14. puberty.ti,ab. (27560)
15. pubescent*.ti,ab. (865)
16. adolescen*.ti,ab. (278039)
17. juvenil*.ti,ab. (81699)
18. underage*.ti,ab. (1211)
19. (preteen* or pre-teen*).ti,ab. (481)
20. (teen or teens or teener).ti,ab. (10684)
21. teenage*.ti,ab. (21165)
22. (youth or youths).ti,ab. (72797)
23. young people*.ti,ab. (28285)
24. young person*.ti,ab. (3499)
25. young wom#n.ti,ab. (30614)
26. (young man or young men).ti,ab. (20422)
27. (highschool or (high adj1 school*)).ti,ab. (32452)
28. sophomore*.ti,ab. (708)
29. (university adj3 student*).ti,ab. (19647)
30. (transition adj4 adult*).ti,ab. (4374)
31. emerging adult*.ti,ab. (2446)
32. young adult*.ti,ab. (94952)
33. early adult*.ti,ab. (7360)
34. freshm?n.ti,ab. (2313)
35. (("10" or "11" or "12" or "13" or "14" or "15" or "16" or "17" or "18" or "19") adj (year* old or year* of age)).ti,ab. (169296)
36. ((ten or eleven or twelve or thirteen or fourteen or fifteen or sixteen or seventeen or eighteen or nineteen) adj (year* old or year* of age)).ti,ab. (4540)
37. (age* adj ("10" or "11" or "12" or "13" or "14" or "15" or "16" or "17" or "18" or "19") adj year*).ti,ab. (36798)
38. (age* adj (ten or eleven or twelve or thirteen or fourteen or fifteen or sixteen or seventeen or eighteen or nineteen) adj year*).ti,ab. (183)
39. or/1-38 (3983043)
40. exp Contraception/ (26828)
41. Family Planning Services/ (24812)
42. exp Contraceptive Devices/ (25273)
43. Contraception Behavior/ (8044)
44. family planning.ti,ab. (21238)
45. contracept*.ti,ab. (67679)
46. ((childbear* or pregnan*) adj2 (avoid* or delay* or prevent* or limit* or space or spacing or timing)).ti,ab. (9890)
47. or/40-46 (116173)
48. Developing Countries/ (74803)
49. ((developing or less* developed or under developed or underdeveloped or middle income or low* income) adj (economy or economies)).ti,ab. (561)
50. ((developing or less* developed or under developed or underdeveloped or middle income or low* income or underserved or under served or deprived or poor*) adj (countr* or nation? or population? or world)).ti,ab. (101164)
51. (low* adj (gdp or gnp or gross domestic or gross national)).ti,ab. (247)
52. (low adj3 middle adj3 countr*).ti,ab. (16855)
53. (lmic or lmics or third world or lami countr*).ti,ab. (7757)
54. transitional countr*.ti,ab. (160)
55. global south.ti,ab. (394)
56. "Democratic People's Republic of Korea"/ (229)
57. (North Korea or (Democratic People* Republic adj2 Korea)).ti,ab. (421)
58. Cambodia/ (3310)
59. Cambodia.ti,ab. (3856)
60. Indonesia/ (10492)
61. (Indonesia or Dutch East Indies).ti,ab. (12412)
62. (Kiribati or Gilbert Islands or Phoenix Islands or Line Islands).ti,ab. (244)
63. Laos/ (1922)
64. (Laos or (Lao adj1 Democratic Republic)).ti,ab. (1966)
65. Micronesia/ (1172)
66. Micronesia.ti,ab. (656)
67. Mongolia/ (1792)
68. Mongolia.ti,ab. (4033)
69. Myanmar/ (2472)
70. (Myanmar or Burma).ti,ab. (4131)
71. Papua New Guinea/ (3453)
72. (Papua New Guinea or German New Guinea or British New Guinea or Territory of Papua).ti,ab. (4504)
73. Philippines/ (8326)
74. (Philippines or Philippine Islands).ti,ab. (8346)
75. Solomon Islands.ti,ab. (805)
76. Timor-Leste/ (204)
77. (Timor-Leste or East Timor or Portuguese Timor).ti,ab. (525)
78. Vanuatu/ (352)
79. (Vanuatu or New Hebrides).ti,ab. (690)
80. Vietnam/ (12258)
81. (Viet Nam or Vietnam or French Indochina).ti,ab. (15137)
82. American Samoa/ (183)
83. American Samoa.ti,ab. (362)
84. exp China/ (193285)
85. China.ti,ab. (180908)
86. Fiji/ (944)
87. Fiji.ti,ab. (1704)
88. Malaysia/ (15038)
89. (Malaysia or Malayan Union or Malaya).ti,ab. (16085)
90. Marshall Islands.ti,ab. (302)
91. Nauru.ti,ab. (153)
92. "Independent State of Samoa"/ (247)
93. ((Samoa not American Samoa) or Western Samoa or Navigator Islands or Samoan Islands).ti,ab. (559)
94. Thailand/ (26407)
95. (Thailand or Siam).ti,ab. (26674)
96. Tonga/ (244)
97. Tonga.ti,ab. (431)
98. (Tuvalu or Ellice Islands).ti,ab. (74)
99. Melanesia/ (1071)
100. Melanesia.ti,ab. (301)
101. Polynesia/ (1873)
102. Polynesia.ti,ab. (1298)
103. Kyrgyzstan/ (1285)
104. (Kyrgyzstan or Kyrgyz Republic or Kirghizia or Kirghiz).ti,ab. (980)
105. Moldova/ (688)
106. Moldova.ti,ab. (515)
107. Ukraine/ (15939)
108. Ukraine.ti,ab. (4675)
109. Uzbekistan/ (1895)
110. Uzbekistan.ti,ab. (1104)
111. Albania/ (839)
112. Albania.ti,ab. (1051)
113. Armenia/ (1408)
114. Armenia.ti,ab. (1044)
115. Azerbaijan/ (1202)
116. Azerbaijan.ti,ab. (1353)
117. "Republic of Belarus"/ (2064)
118. (Belarus or Byelarus or Byelorussia or Belorussia).ti,ab. (1543)
119. Bosnia-Herzegovina/ (2121)
120. (Bosnia or Herzegovina).ti,ab. (2317)
121. Bulgaria/ (6358)
122. Bulgaria.ti,ab. (4189)
123. "Georgia (Republic)"/ (1802)
124. Georgia.ti,ab. not Georgia/ (5960)
125. Kazakhstan/ (2665)
126. (Kazakhstan or Kazakh).ti,ab. (2743)
127. Kosovo/ (202)
128. Kosovo.ti,ab. (923)
129. Montenegro/ (214)
130. Montenegro.ti,ab. (823)
131. "Republic of North Macedonia"/ (557)
132. North Macedonia.ti,ab. (55)
133. Romania/ (10034)
134. Romania.ti,ab. (5512)
135. exp Russia/ (53208)
136. "Russia (Pre-1917)"/ (5981)
137. USSR/ (42765)
138. (Russia or Russian Federation or USSR or Union of Soviet Socialist Republics or Soviet Union).ti,ab. (28150)
139. Serbia/ (3133)
140. Serbia.ti,ab. (4315)
141. Turkey/ (34585)
142. (Turkey.ti,ab. not animal/) or (Anatolia or Asia Minor).ti,ab. (25104)
143. Turkmenistan/ (576)
144. Turkmenistan.ti,ab. (343)
145. Tajikistan/ (741)
146. Tajikistan.ti,ab. (580)
147. Asia, Central/ (475)
148. Asia, Northern/ (20)
149. Central Asia.ti,ab. (2269)
150. Haiti/ (3156)
151. (Haiti or Hayti).ti,ab. (3035)
152. Bolivia/ (2571)
153. Bolivia.ti,ab. (3228)
154. El Salvador/ (871)
155. El Salvador.ti,ab. (1237)
156. Honduras/ (1119)
157. Honduras.ti,ab. (1737)
158. Nicaragua/ (1480)
159. Nicaragua.ti,ab. (1852)
160. Argentina/ (15692)
161. (Argentina or Argentine Republic).ti,ab. (16531)
162. Belize/ (576)
163. (Belize or British Honduras).ti,ab. (843)
164. Brazil/ (93168)
165. Brazil.ti,ab. (82703)
166. Colombia/ (10376)
167. Colombia.ti,ab. (12026)
168. Costa Rica/ (3662)
169. Costa Rica.ti,ab. (4837)
170. Cuba/ (5016)
171. Cuba.ti,ab. (4477)
172. Dominica/ (98)
173. Dominica.ti,ab. (472)
174. Dominican Republic/ (1561)
175. Dominican Republic.ti,ab. (1887)
176. Ecuador/ (3711)
177. Ecuador.ti,ab. (4468)
178. Grenada/ (142)
179. Grenada.ti,ab. (314)
180. Guatemala/ (2966)
181. Guatemala.ti,ab. (3500)
182. Guyana/ (683)
183. (Guyana or British Guiana).ti,ab. (1080)
184. Jamaica/ (3426)
185. Jamaica.ti,ab. (3226)
186. Mexico/ (38352)
187. (Mexico or United Mexican States).ti,ab. (41958)
188. Paraguay/ (786)
189. Paraguay.mp. (1678)
190. Peru/ (8735)
191. Peru.ti,ab. (10340)
192. Saint Lucia/ (69)
193. (St Lucia or Saint Lucia or Iyonala or Hewanorra).ti,ab. (339)
194. "Saint Vincent and the Grenadines"/ (52)
195. (Saint Vincent or St Vincent or Grenadines).ti,ab. (603)
196. Suriname/ (927)
197. (Suriname or Dutch Guiana).ti,ab. (572)
198. Venezuela/ (4896)
199. Venezuela.ti,ab. (5227)
200. Djibouti/ (226)
201. (Djibouti or French Somaliland).ti,ab. (384)
202. Egypt/ (14699)
203. Egypt.ti,ab. (13915)
204. Morocco/ (5673)
205. Morocco.ti,ab. (5460)
206. Tunisia/ (8275)
207. Tunisia.mp. (10358)
208. (Gaza or West Bank or Palestine).ti,ab. (2434)
209. Algeria/ (3040)
210. Algeria.ti,ab. (3189)
211. Iran/ (26728)
212. (Iran or Persia).ti,ab. (37869)
213. Iraq/ (4619)
214. (Iraq or Mesopotamia).ti,ab. (6991)
215. Jordan/ (4207)
216. Jordan.ti,ab. (6109)
217. Lebanon/ (4260)
218. (Lebanon or Lebanese Republic).ti,ab. (4462)
219. Libya/ (1120)
220. Libya.ti,ab. (1250)
221. Syria/ (1810)
222. (Syria or Syrian Arab Republic).ti,ab. (1994)
223. Yemen/ (1381)
224. Yemen.ti,ab. (1814)
225. Afghanistan/ (3197)
226. Afghanistan.ti,ab. (5834)
227. Nepal/ (8128)
228. Nepal.ti,ab. (9629)
229. Bangladesh/ (10942)
230. Bangladesh.ti,ab. (13312)
231. Bhutan/ (458)
232. Bhutan.ti,ab. (731)
233. exp India/ (102909)
234. India.ti,ab. (97774)
235. Pakistan/ (17537)
236. Pakistan.ti,ab. (17947)
237. Maldives.ti,ab. (330)
238. Sri Lanka/ (5993)
239. (Sri Lanka or Ceylon).ti,ab. (6894)
240. Angola/ (997)
241. Angola.ti,ab. (1388)
242. Cameroon/ (5461)
243. (Cameroon or Kamerun or Cameroun).ti,ab. (6869)
244. Cape Verde/ (199)
245. (Cape Verde or Cabo Verde).ti,ab. (598)
246. Comoros/ (307)
247. (Comoros or Glorioso Islands or Mayotte).ti,ab. (554)
248. Congo/ (1848)
249. (Congo not ((Democratic Republic adj3 Congo) or congo red or crimean-congo)).ti,ab. (2549)
250. Cote d'Ivoire/ (3114)
251. (Cote d'Ivoire or Cote dIvoire or Ivory Coast).ti,ab. (3806)
252. Eswatini/ (579)
253. (eSwatini or Swaziland).ti,ab. (912)
254. Ghana/ (8167)
255. (Ghana or Gold Coast).ti,ab. (10613)
256. Kenya/ (15935)
257. (Kenya or East Africa Protectorate).ti,ab. (17819)
258. Lesotho/ (420)
259. (Lesotho or Basutoland).ti,ab. (704)
260. Mauritania/ (441)
261. Mauritania.ti,ab. (617)
262. Nigeria/ (28351)
263. Nigeria.ti,ab. (28272)
264. (Sao Tome adj2 Principe).ti,ab. (151)
265. Senegal/ (5694)
266. Senegal.ti,ab. (5639)
267. Sudan/ (4684)
268. (Sudan not South Sudan).ti,ab. (7349)
269. Zambia/ (4496)
270. (Zambia or Northern Rhodesia).ti,ab. (5215)
271. Zimbabwe/ (5793)
272. (Zimbabwe or Southern Rhodesia).ti,ab. (5620)
273. Botswana/ (1786)
274. (Botswana or Bechuanaland or Kalahari).ti,ab. (2549)
275. Equatorial Guinea/ (265)
276. (Equatorial Guinea or Spanish Guinea).ti,ab. (424)
277. Gabon/ (1449)
278. (Gabon or Gabonese Republic).ti,ab. (1722)
279. Mauritius/ (562)
280. (Mauritius or Agalega Islands).ti,ab. (967)
281. Namibia/ (1074)
282. (Namibia or German South West Africa).ti,ab. (1507)
283. South Africa/ (41839)
284. (South Africa or Cape Colony or British Bechuanaland or Boer Republics or Zululand or Transvaal or Natalia Republic or Orange Free State).ti,ab. (33743)
285. Benin/ (1539)
286. (Benin or Dahomey).ti,ab. (3401)
287. Burkina Faso/ (3219)
288. (Burkina Faso or Burkina Fasso or Upper Volta).ti,ab. (4184)
289. Burundi/ (634)
290. (Burundi or Ruanda-Urundi).ti,ab. (884)
291. Central African Republic/ (778)
292. (Central African Republic or Ubangi-Shari).ti,ab. (1014)
293. Chad/ (718)
294. Chad.ti,ab. (1153)
295. "Democratic Republic of the Congo"/ (4186)
296. (((Democratic Republic or DR) adj2 Congo) or Congo-Kinshasa or Belgian Congo or Zaire or Congo Free State).ti,ab. (4465)
297. Eritrea/ (345)
298. Eritrea.ti,ab. (536)
299. Ethiopia/ (12687)
300. (Ethiopia or Abyssinia).ti,ab. (15414)
301. Gambia/ (2407)
302. Gambia.ti,ab. (2290)
303. Guinea/ (1036)
304. (Guinea not (New Guinea or Guinea Pig* or Guinea Fowl or Guinea-Bissau or Portuguese Guinea or Equatorial Guinea)).ti,ab. (2608)
305. Guinea-Bissau/ (925)
306. (Guinea-Bissau or Portuguese Guinea).ti,ab. (1022)
307. Liberia/ (1204)
308. Liberia.ti,ab. (1541)
309. Madagascar/ (3421)
310. (Madagascar or Malagasy Republic).ti,ab. (4712)
311. Malawi/ (5263)
312. (Malawi or Nyasaland).ti,ab. (6875)
313. Mali/ (2331)
314. Mali.ti,ab. (3471)
315. Mozambique/ (2393)
316. (Mozambique or Mocambique or Portuguese East Africa).ti,ab. (3567)
317. Niger/ (1186)
318. (Niger not (Aspergillus or Peptococcus or Schizothorax or Cruciferae or Gobius or Lasius or Agelastes or Melanosuchus or radish or Parastromateus or Orius or Apergillus or Parastromateus or Stomoxys)).ti,ab. (3410)
319. Rwanda/ (2407)
320. (Rwanda or Ruanda).ti,ab. (2980)
321. Sierra Leone/ (1516)
322. (Sierra Leone or Salone).ti,ab. (2209)
323. Somalia/ (1581)
324. (Somalia or Somaliland).ti,ab. (1476)
325. South Sudan/ (149)
326. South Sudan.ti,ab. (528)
327. Tanzania/ (11298)
328. (Tanzania or Tanganyika or Zanzibar).ti,ab. (13390)
329. Togo/ (1133)
330. (Togo or Togolese Republic or Togoland).ti,ab. (1459)
331. Uganda/ (12017)
332. Uganda.ti,ab. (14085)
333. "africa south of the sahara"/ (11035)
334. africa, central/ (1278)
335. africa, eastern/ (4070)
336. africa, southern/ (2373)
337. africa, western/ (5817)
338. ("Africa South of the Sahara" or sub-Saharan Africa or subSaharan Africa).ti,ab. (21003)
339. Central Africa.ti,ab. (3108)
340. Eastern Africa.ti,ab. (975)
341. Southern Africa.ti,ab. (4279)
342. Western Africa.ti,ab. (831)
343. or/48-342 (1488989)
344. 39 and 47 and 343 (16244)
345. limit 344 to yr="2016 -Current" (2845)
346. limit 345 to (english or portuguese) (2792)
