# Supplementary material for: Can cash transfer interventions increase contraceptive use and reduce adolescent birth and pregnancy in low and middle income countries? A systematic review and meta-analysis
Source: PLOS Glob Public Health. 2023 Nov 9;3(11):e0001631. doi: 10.1371/journal.pgph.0001631 (PMC10635429; doi:10.1371/journal.pgph.0001631)
Supplement: S2 Appendix — (DOCX) [file pgph.0001631.s003.docx]

# Appendix 2: Characteristics of studies

| **Study (Author, Year, Location)** | **Intervention name and description** | **Study Design, Study Period and Study Size** | **Relevant Outcome measures for this study and age range at start and end** | **Main Findings (reported by author)** | **Risk of bias and tool** |
| --- | --- | --- | --- | --- | --- |
| Alam, Baez (1)  Punjab, Pakistan | **Intervention name**  Punjab Female School Stipend Program  **Intervention description**  **Conditional Cash Transfer:** The main goal was to promote participation in public education for girls in middle school. Program included a quarterly stipend of approximately PKR 600 (equivalent to US$10) per student. Girls were targeted based on their district of residence (districts with the lowest literacy rates in Punjab). | **Study design**  **Quasi-experimenta**l: the analysis contrasts cross-sectional data at two time points and contrasts girls living in areas where the stipend was delivered with girls living in control districts. The analysis employs double-difference and regression discontinuity design frameworks. Girls were targeted by their district of residence: only districts with a literacy rate below 40% received the treatment (literacy in Punjab, 2003: 54%).  **Study period**  Study compared data collected pre-programme (2003) and post-programme (2007-8)  **Study size**  Compares outcomes of 392 girls with respect to fertility. | **Relevant outcomes:**  Mean change in probability of giving birth aged 17-19  **Age range:**  Intervention directed at middle school – when girls aged 10-12 years | The “analysis reveals no differential effects on the probability of girls 17-19 having given birth” | **CASP Cohort Study checklist:**  Study deemed to have minor concerns.  Concerns around the comparability of interventions and attribution of any impacts.  We do not know how far from the 40% cut-off either group of districts were (this being esp. concerning for the RDD). |
| Austrian, Soler-Hampejsek (2)  Kiberia (Nairobi) and Wajir, Kenya | **Intervention name**  Adolescent Girls Initiative–Kenya  **Intervention description**  **Conditional Cash Transfer:** A cash transfer was provided to eligible girls conditioned on school enrollment and regular attendance. The four components of the conditional transfer included: 1) fees, if applicable, to be paid directly to the school; 2) a cash transfer paid to the head of the household; 3) schooling kits given directly to girls at the start of each term; and 4) incentive paid directly to the school based on the number of girls enrolled in the cash transfer program. This intervention took place alongside a violence prevention intervention which girls in the control arm areas were also exposed to – the violence prevention intervention consisted of communities identifying strategies to reduce violence against women and girls. Additional elements in one arm included facilitated discussions on health; in another this included facilitated discussion and a wealth creation/financial literacy intervention. All intervention arms included a CCT | **Study design**  **RCT and CRCT**: RCT with active control (violence prevention). The unit of randomisation was individual in Kiberia and population clusters in Wajir.  **Study period**  The interventions were delivered between 2015 and 2017 and endline data collection took place in 2019  **Study size**  The outcomes of 1012 girls were analysed at endline for the two intervention arms of interest. | **Relevant outcomes:**  Post-test - Whether girls had been pregnant, given birth, or had used modern family planning methods.  **Age range:**  The primary target population was girls ages 11–14 at the time of the baseline survey (and 13-18 | In Wajir, ‘the CCT led to long-term improvements in delayed …pregnancy (34% v. 17%)’ and in Kiberia ‘the CCT had an impact on delaying ….. pregnancy by … 43%’. Other results are either not significantly different or are not reported. | **CASP RCT Study checklist:**  No major concerns |
| Baird, Garfein (3)  Baird, McIntosh (4)  Malawi | **Intervention name**  Zomba Cash Transfer Program  **Intervention description**  **Unconditional and Conditional Cash Transfer:** One group received conditional cash transfers (CCT) and the other unconditional cash transfers (UCT). Participants in the CCT arm received monthly cash transfers conditional on regular school attendance. The UCT arm also received transfers except school attendance was not required. Participants in the control group received nothing. | **Study design**  **CRCT**: Cluster-randomized controlled trial with two active arms.  **Study period**  The intervention began in early 2008 and continued through the end of 2009. Follow up surveys were conducted in 2010, and 2012. Data from 2010 analysed in this study.  **Study size**  2,907 were in school at baseline with mean age of 15; an alternative group who had dropped out of school numbered 889. | **Relevant outcomes:**  Post-test – Currently pregnant; Ever pregnant.  (Note – no indicator of contraception use besides condom use)  **Age range:**  Mean age 15 at baseline | “The adjusted point estimates for the likelihood of … pregnancy… did not differ significantly between intervention and control groups. | **CASP RCT Study checklist:**  No major concerns |
| Barham, Macours (5)  Nicaragua | **Intervention name**  Red de Protección Social (RPS)  **Intervention description**  **Conditional Cash Transfer:** The CCT had two core components. The first core component focused on nutrition and health. In the second core component, households with children ages 7–13 years who had not yet completed the fourth grade of primary school were eligible for a fixed bimonthly cash transfer known as the school attendance transfer, which was contingent on school enrollment and attendance; households also received an additional annual cash transfer for school supplies, contingent on school enrolment. | **Study design**  **CRCT**: Cluster-randomized controlled trial with 42 localities randomised into two different groups – one group who receiving the intervention earlier and one later  **Study period**  Randomisation occurred in 2000; the early recipients received the CCT from 2000-2003; the late recipients 2003-2005. All had received the CCT when the outcome was assessed  **Study size**  Data from 14,104 were collected on births at 10 year follow-up | **Relevant outcomes:**  Post-test – Had live birth  (***Note – data not used in meta-analysis for comparability as both groups received full intervention when outcome assessed***)  **Age range:**  Participants were aged 19-22 at follow-up | “Differential exposure to the CCT … does lead to differential impacts on the age of menarche, young adult BMI, fertility, and subsequent labor market outcomes and income.” | **CASP RCT Study checklist:**  No concerns with study design, although concern about comparability with other studies as all participants had received the intervention |
| Buchmann, Field (6)  Buchmann, Field (7)  Bangladesh | **Intervention name**  Jibon-O-Jibika Program (with Kishoree Kontha (KK), or “Adolescent Girl’s Voice”)  **Intervention description**  **Conditional Cash Transfer:** The CCT was an “in-kind transfer of cooking oil to encourage parents to postpone daughters’ marriage until the legal age of consent (18 years). The value of the incentive was approx. $16 per year, an amount similar to the cost of higher dowry. Cooking oil was chosen because it is purchased regularly by every family in Bangladesh and thus has close to cash equivalent value, yet it is less susceptible to theft and graft than cash because of its bulk. Only girls (not their parents) were permitted to collect the oil by presenting their ration card. The intervention was offered standalone and alongside an empowerment intervention – the Kishoree Kontha” | **Study design**  **CRCT**: Cluster-randomized controlled trial where 460 communities in 5 sub-districts were randomised into either a CCT arm, a CCT arm plus an empowerment intervention, an empowerment intervention alone, or usual care.  **Study period**  The trial started in 2007. All had received the CCT when the outcome was assessed.  **Study size**  Data were collected at follow-up from a total of 2,349 participants in the conditional incentive arm, and 2,659 in the empowerment plus incentive arm; 5,337 participants were included in the control arm. | **Relevant outcomes:**  Post-test – Teenage childbearing – under 20  **Age range:**  Girls were aged 15-17 at the beginning of the trial | “Data from girls 4.5 years after program completion show that girls eligible for the incentive for at least two years were 14% (-5.2ppts, p<0.01) less likely to have given birth under 20.” “The incentive reduced the likelihood of teenage childbearing by 11% (-2.5ppts, p<0.05). We again do not observe a separate or additional effect of the empowerment program.” | **CASP RCT Study checklist:**  No concerns |
| Dake, Natali (8)  Malawi | **Intervention name**  Social Cash Transfer Program (SCTP)  **Intervention description**  **Unconditional Cash Transfer:** The SCTP offers support to the most vulnerable households and aims to relieve poverty and improve school enrolment. | **Study design**  **CRCT**: Cluster randomized-controlled trial (cRCT); 29 villages (14 assigned to the treatment arm through public lottery).  **Study period**  The evaluation was conducted in 2013-2015, 30 months after the programme had been initiated.  **Study size**  Data were collected from 917 female participants at endline | **Relevant outcomes:**  Post-test – Ever pregnant (girls aged 17-23 years); Currently pregnant (girls aged 17-23 years)  *Note used data on ever pregnant  **Age range:**  Girls were aged 14-21 at baseline (mean 16.4) | “After two and three years of transfers, there are few measurable impacts on safe transition outcomes for males or females” | **CASP RCT Study checklist:**  No concerns |
| Dake, Natali (8)  Zambia | **Intervention name**  Multiple Categorical Targeted Grant (MCTG)  **Intervention description**  **Unconditional Cash Transfer:** The MCTG is an UCT aimed at vulnerable households with an aim of reducing the risk of intergenerational poverty. | **Study design**  **CRCT**: Cluster randomized-controlled trial (cRCT); 92 communities (46 assigned to the treatment arm through public lottery)  **Study period**  The evaluation was conducted in 2011-2013, 36 months after the programme had been initiated.  **Study size**  Data were collected from 1210 female participants at endline | **Relevant outcomes:**  Post-test – Ever pregnant (girls aged 17-23 years); Currently pregnant (girls aged 17-23 years)  *Note used data on ever pregnant  **Age range:**  Girls were aged 14-21 at baseline **(mea** | “After two and three years of transfers, there are few measurable impacts on safe transition outcomes for males or females” | **CASP RCT Study checklist:**  Baseline differences in gender progressiveness and age impeded on interpretation |
| Darney, Weaver (9)  Mexico | **Intervention name**  Oportunidades - established by the Mexican government in 1997 as PROGRESA  **Intervention description**  **Conditional Cash Transfer: ‘**Provides money to female household heads or wives of household heads contingent on household compliance with gender- and age-specific health service utilisation requirements, such as prenatal, postpartum and pediatric visits, as well as nutritional supplementation and school attendance. Amount equals approximately 20% of the family’s pre-program monthly expenditures.’ | **Study design**  **Quasi-experimental**: Cross-sectional data on outcomes with indicator of exposure to programme  **Study period**  Data collected in 2006, 9 years after programme started; length of exposure of individual households unclear  **Study size**  Data were collected from 2,034 female participants aged 15-19 in 2006 | **Relevant outcomes:**  Post-test – Ever pregnant; Currently pregnant; Currently using any contraceptive methods  **Age range:**  Data for girls aged 15-19 available | “ We found no evidence that Oportunidades had a direct effect on pregnancy among adolescents or on current modern contraceptive use among adolescent and young adult women” | **CASP non-RCT Study checklist:**  No longitudinal component at the individual level; length of exposure unclear |
| Dunbar, Kang Dufour (10)  Zimbabwe | **Intervention name**  Shaping the Health of Adolescents in Zimbabwe (SHAZ!)  **Intervention description**  **Conditional Cash Transfer: ‘**Randomized controlled trial of a combined intervention package including life-skills and health education, vocational training, micro-grants and social supports compared to life-skills and health education alone. Participants who successfully completed a vocational training developed business plans that were supported with a micro-grant in the form of capital equipment, supplies or additional training. The resources purchased with these micro-grants were valued at $100 US dollars or less per participant.’ The mentioning is described as supporting the livelihoods training and micro-grants. Active control arm – all participants received life skills training and reproductive health services  Note: (1) the study purposively recruited HIV uninfected adolescent orphans; (2) receipt of transfer was conditional on developing business plan (selection effects possible). | **Study design**  **RCT**: RCT with individuals randomised  **Study period**  Enrolment in 2006 programme and data collection started at baseline with the endline results collected 24 months afterwards  **Study size**  158 participants involved although only 58% received the micro-grant. | **Relevant outcomes:**  Post-test – Contraceptive use with current partner; Unintended pregnancy  **Age range:**  The average age at the start of the intervention was 18 years and data collected two years after | “There was evidence of fewer unintended pregnancies among intervention participants [HR50.61, 95% CI (0.37, 1.01)], although this relationship achieved only marginal statistical significance…. No statistically significant changes were reported for contraceptive use” | **CASP RCT Study checklist:**  No concerns |
| Erulkar and Muthengi (11)  Ethiopia | **Intervention name**  Berhane Hewan: A Program To Delay Child Marriage in Rural Ethiopia  **Intervention description**  **Conditional Cash Transfer: ‘**  ‘Intervention consisted of support groups to encourage girls to stay in school. Girls in support groups received approx. $4 in school materials. After eligible girls were invited to participate, their parents or guardians were asked for permission.  Parents of unmarried girls agreed that their daughters would not be married during the two-year programme; they also agreed to allow them to attend the programme meetings. Parents were promised that if they did not arrange to marry their daughters during this period, and their daughters attended at least 80% of the group sessions, a goat would be presented jointly to the girl and her family at the end of the program. At graduation, goats were worth about 180 birr (US$20).’ | **Study design**  **Quasi-experimental study**: Baseline and endline surveys in intervention and matched control village.  **Study period**  Baseline survey conducted in 2004; endline survey conducted in 2006  **Study size**  Unclear – 668 girls interviewed at endline in intervention villages and approximately 73-85% had taken part in intervention components; number of participants at endline considerably higher than baseline. | **Relevant outcomes:**  Post-test – Ever used contraceptive methods  **Age range:**  Girls aged 10-19 with 57% aged 10-14 at the start of the project. | “The intervention was associated with considerable improvements in girl’s school enrollment, age at marriage, reproductive health knowledge and contraceptive use.” | **CASP non-RCT Study checklist:**  Major concerns - Not longitudinal at individual level and many interviewed at follow-up may not have received the intervention; concerns about confounding. |
| Handa, Peterman (12)  Kenya | **Intervention name**  Kenya Cash Transfer for Orphans and Vulnerable Children  **Intervention description**  **Unconditional Cash Transfer: ‘**  ‘Unconditional cash transfer (UCT), meaning that receipt of cash is not tied to any specific behavior or compliance of the household. Households were eligible where they were found to be ‘ultra’ poor and with an elderly head of household. The sum comprised approx. 20% of total monthly household expenditure.’ | **Study design**  **CRCT**: Cluster randomised controlled trial with wait list control  **Study period**  The intervention was implemented 2007 and endline data collected in 2011  **Study size**  Approx 1,542 and 755 treatment and control households respectively | **Relevant outcomes:**  Post-test – Ever been pregnant  **Age range:**  Data collected from 1547 girls at endline with mean age of 16. | “Findings indicate that, among 1,549 females included in the study, while the program reduced the likelihood of pregnancy by five percentage points, there was no significant impact on likelihood of early marriage.” | **CASP non-RCT Study checklist:**  Minor concerns about baseline equivalence between recipient and control households in which girls live |
| Martínez-Restrepo (13)  Brazil | **Intervention name**  Young Agent Project in Brazil (Projeto Agente Jovem)  **Intervention description**  **Conditional Cash Transfer: ‘**  ‘To receive the YAP cash transfer, adolescents must attend school and a federal funded after school program at least 80 percent of the time. In 2009, the cash transfer was R$65 (approximately US$33) per month per adolescent enrolled in the program. A feature of the YAP is that the cash transfer can be received directly by the adolescent, their parents, the legal guardian or the head of the household’ | **Study design**  **Quasi-experimental**: The data used is survey data that include a matched non-experimental comparison group.  **Study period**  Survey participants received the CCT between 2002 and 2005; the survey was conducted in 2006  **Study size**  The main sample used consisted of a control of 495 and a treatment group of 1,688 adolescents | **Relevant outcomes:**  Post-test – Use of contraceptive methods and experience of teen pregnancy  **Age range:**  Adolescents were aged 15-17 on receipt of the CCT and the survey of former beneficiaries included those aged 16-20 years. | “Regarding risky behaviors, this dissertation finds increased use of contraceptives among the treatment group. Although, there were no reductions in teen pregnancy, the higher use of contraceptives is consistent with reductions in unplanned pregnancies among females and the 18 – 20 age subgroup.” | **CASP non-RCT Study checklist:**  Cross-sectional approach therefore weaker study design - minor concern about reporting of response rates to questions and number of matches |
| MercyCorps (14)  Niger | **Intervention name**  Sawki programme  **Intervention description**  **Conditional Cash Transfer: ‘**  ‘Intervention is deemed eligible as participants received sizable and transferable incentive for participation (50kg bag of lentils). Participation involved taking part on one of two arms: ‘Safe Space (SS) model (35 hours of direct intervention over 8 months) focuses on teaching girls essential nutrition actions, risks associated with early marriage and early pregnancy, reproductive health and the importance of education and basic literacy. The Safe Space + Livelihood (SS+L) model (87 to 91 hours of intervention over 19 to 20 months) includes all Safe Space components, but adds livelihood trainings that focus on livestock management (i.e., goat production, poultry care, and animal health training); gardening activities; and savings and loans activities’ | **Study design**  **Quasi-experimental**: The researchers implemented a propensity score matching (PSM) difference-in-difference model to match girls within the two distinct treatment groups with a separate sample of girls in the comparison village group.  **Study period**  The survey was conducted in early 2015 by which point participants had been exposed to an 8 month intervention (SS) or an 18 month intervention (SSL)  **Study size**  Data were collected on 394 girls in the control group, 207 in SS, and 228 in SSL. | **Relevant outcomes:**  Mean change – Contraceptive use  **Age range:**  Adolescents were aged 10-18 with a mean age of 14 | “The findings suggest that neither the SS nor the SS+L have increased the likelihood that girls are using contraceptives or their access.” | **CASP non-RCT Study checklist:**  Minor concerns – lack of information hinders assessing full quality although doubts on success of matching |
| Özler, Hallman (15)  Liberia | **Intervention name**  Girl Empower  **Intervention description**  **Conditional Cash Transfer: ‘**  ‘The GE intervention delivered a life skills curriculum to girls aged 13–14 in Liberia, facilitated by local female mentors. All participating girls received cash to help start their own savings account, along with a savings book and a cash box. Each girl received $2 per month for a total of $16 during the eight-month implementation period. In a different arm (GE+), in addition to further sessions, caregivers of program participants received of a payment of $1.25 for each of the 32 regular sessions that the adolescent girl attended up to a maximum of $40’ | **Study design**  **CRCT**: The researchers conducted a parallel cluster-randomized controlled trial with three arms: control, GE, and GE+ (allocation ratio: 1:1:1)  **Study period**  Registration for the intervention started in late 2015. Follow up data collection started in 2017 and continued until 2018 (approx. 2 years later).  **Study size**  Data were collected on 383 girls in the control group, 393 in GE, and 400 in GE+ | **Relevant outcomes:**  Post-test – (Never) Pregnant  **Age range:**  Adolescents were aged 13-14 at the start of the intervention | “Neither variant of GE had a detectable effect on age at first marriage and pregnancy, and labour market participation” | **CASP RCT Study checklist:**  No major concerns |
| Rosenberg, Bhushan (16)  Malawi | **Intervention name**  Girl Power Malawi  **Intervention description**  **Conditional Cash Transfer:** A CCT was provided in conjunction with an integrated Youth Focussed Health Space (YFHS). In addition a behavioural intervention delivered in a number of sessions was implemented that supported adolescents with life and problem solving skills (BI). The CCT involved participants receiving a monthly transfer of $5.50 for attending the YFHS and the BI. | **Study design**  **Quasi-experimental**: Four health centres offered one of four arms (random allocation). ES based on comparing cash transfer arm to control.  **Study period**  The intervention was conducted Feb 2016-Aug 2017 and participants submitted evaluation data at 6 and 12 months after baseline.  **Study size**  Each heath centre recruited 250 girls | **Relevant outcomes:**  Post-test – Uptake of hormonal contraception and intrauterine contraception  **Age range:**  Adolescents were aged 15-24 years old | “These findings demonstrate that offering adolescents a model of YFHS service delivery, which includes youth-focused spaces, young peer educators, youth-friendly health providers, and integrated services leads to considerably higher uptake of contraceptives” | **CASP RCT Study checklist:**  Concerns about substantial baseline differences in pregnancy and marital histories |
| Stecklov, Winters (17)  Mexico  Honduras  Nicaragua | **Intervention name**  Education, Health and Nutrition Program- Progresa (Mexico)  Family Assistance Program (PRAF) (Honduras)  Social Protection Network (RPS) (Nicaragua)  **Intervention description**  **Conditional Cash Transfer:** CCTs were provided with difference sets of requirements. In Mexico, these were estimated to be equivalent to 20% of consumption value; in Nicaragua 21%; in Honduras this was estimated to be lower (value not provided) | **Study design**  **CRCT**: communities were randomly assigned to treatment and control groups  **Study period**  ‘To date, a total of two rounds of survey data have been collected for PRAF (2000 and 2002), three rounds for RPS (2000 and 2001, and 2002), and six rounds for PROGRESA (two each in 1998, 1999 and 2000) along with a baseline census in 1997.’  **Study size**  Over 5000 households involved in each side, although numbers of adolescent girls varied | **Relevant outcomes:**  Post-test and mean change – Fertility data available  (***Note – data not used in meta-analysis for comparability as accurate data disaggregated by age is not presented***) | N/A – not applicable – findings not reported in main analysis | **CASP RCT Study checklist:**  N/A– not applicable – findings not reported in main analysis |

# Quality assessment tables – CASP cohort

| Author (date) | Did the study address a clearly focused issue? | Was the cohort recruited in an acceptable way? | Was the exposure measured to minimise bias? | Was the outcome measured to minimise bias? | Have important confounding factors been identified? | Have confounding factors been taken account of in the analysis? | Was the follow-up of subjects complete enough? | Overall assessment of concerns |
| --- | --- | --- | --- | --- | --- | --- | --- | --- |
| Alam, Baez (1) | Yes – explore impacts of a gender targeted CCT in the longer term | Can’t tell - area based intervention but nothing about coverage within areas | Can’t tell - Ascertainment based on current residence not on actual exposure to intervention | Can’t tell – unclear if girls were sexually active | Yes - They use ‘a comprehensive set of social and economic indicators as well as information on infrastructure supply and quality, allowing for construction of some covariates at the district level’. | Yes – Authors claim that their techniques deal with potential biases that may rise due to area-based differences | No – this was repeated cross-sectional analysis looking at area-level changes | Minor concerns – Attribution of impacts given that this is a repeated cross-sectional design comparing districts that have been targeted in very different ways. |
| Darney, Weaver (9) | Yes | Yes | Yes – although length of exposure unclear | Can’t tell – unclear if girls were sexually active | Can’t tell | Can’t tell | Can’t tell | Major concerns - No longitudinal component at the individual level; length of exposure unclear; details of clustering unclear |
| Erulkar and Muthengi (11) | Yes | Unclear – it was intended that three villages would be involved in the intervention, although two dropped out | No – dependent on recall at post-test; estimated that only 73-85 of eligible participants had taken part in intervention. In addition, sample at endline is much larger than baseline. | Yes – data pertains to sexually active girls | No | No | No – this was repeated cross-sectional analysis looking at area-level changes | Major concerns - Not longitudinal at individual level and many interviewed at follow-up may not have received the intervention; concerns about confounding. |
| Martínez-Restrepo (13) | Yes | Yes | Yes | Yes – but question not described | Can’t tell – similarity of groups | Can’t tell – number of appropriate matches unclear in the data | Can’t tell | Minor concern about response rates to questions and number of matches |
| MercyCorps (14) | Yes | Unclear – little information provided on recruitment | Can’t tell – other elements of broader Sawki intervention had been implemented across intervention and control villages | Yes – data pertains to married girls; authors claim that sexual activity almost exclusively within marriage | Can’t tell | Unclear - Heterogeneity across treatment groups for age, marital status, school enrolment, and educational qualification (reading); does matching really account for (community level) confounders? | Unclear – different follow up times for different arms | Minor concerns – lack of information hinders assessing full quality although doubts on success of matching |

# Quality assessment tables – CASP RCT

| Author (date) | Did the study address a clearly focused research question | Was the assignment of participants to interventions randomised? | Were all participants who entered the study accounted for at its conclusion? | Were the participants ‘blind’ to intervention they were given? | Were the investigators ‘blind’ to the intervention they were giving to participants? | Were the people assessing/ analysing outcome/s ‘blinded’? | Were the study groups similar at the start of the randomised controlled trial? | Apart from the experimental intervention, did each study group receive the same level of care (that is, were they treated equally)? | Were the effects of intervention reported comprehensively? | Was the precision of the estimate of the intervention or treatment effect reported? | Do the benefits of the experimental intervention outweigh the harms and costs? |
| --- | --- | --- | --- | --- | --- | --- | --- | --- | --- | --- | --- |
| Austrian, Soler-Hampejsek (2) | Yes – explore impacts of a series of multi-sectoral interventions | Yes | Yes - an intent to treat (ITT) approach was followed. | No | No | No | Yes – they were broadly similar | Yes | Yes | Can’t tell – descriptive data only | Yes, mixed methods allowed girls to report on adverse outcomes if applicable |
| Baird, Garfein (3) | Yes – examining the eﬃcacy of a cash transfer programme. | Yes – through computer-generated list of random numbers | Yes – primary outcomes assessed by ITT analysis | Can’t tell - Study participants were not masked to assignment, but did not know what the comparison groups were | No | No | Yes – they were broadly similar | Yes | Yes | Yes | Can't tell with respect to pregnancy; consideration of harms missing |
| Barham, Macours (5) | Yes – focused on long-term follow-up of intervention | Yes | Yes – ITT estimates provided | No | No | No | Yes – they were broadly similar | Yes – although long follow-up and other area based policies could have been implemented | Yes | Yes | Yes |
| Buchmann, Field (6)  Buchmann, Field (7) | Yes | Yes | Yes – in part ITT estimates were provided | No | No | No | Yes - minor differences in the contraceptive knowledge in one arm | Yes | Yes | Yes | Yes |
| Dake, Natali (8)  Malawi | Yes | Yes | Yes – ITT | No | No | No | Yes | Yes | Yes | Yes | No – the intervention appears to accelerate transitions to parenthood and pregnancy in Malawi |
| Dake, Natali (8)  Zambia | Yes | Yes | Yes – ITT | No | No | No | Can’t tell – differences in age, school attendance and gender progressiveness in Zambia | Yes | Yes | Yes | Can’t tell – no apparent effect on fertility but some differences in education |
| Dunbar, Kang Dufour (10) | Yes | Yes | Yes – ITT | No | No | No | No – higher levels of secondary education in the treatment group | Yes | Yes | Yes | Can’t tell – no apparent effect on fertility or unwanted pregnancy although the latter may be underpowered |
| Handa, Peterman (12) | Yes | Yes – although trialists note that this was not as expected | Can’t tell – attrition levels were high due to election turmoil and unclear how impacted on analyses | No | No | No | No – differences in household characteristics | Yes | Yes | Yes | Can’t tell – appears to be a difference in the age, educational status, and gender of head of household between treatment and control at baseline |
| Özler, Hallman (15) | Yes | Yes | Yes – ITT | No | No | No | Yes | Yes | Yes | Yes | Can’t tell – little observable effect on fertility |
| Rosenberg, Bhushan (16) | Yes | Yes | Yes – ITT | No | No | No | No – important differences in Marital and Pregnancy histories and age | Yes | No – adjusted estimates stratified by arm not clearly reported across outcomes | Can’t tell | Can’t tell – appears to be a substantial impact on hormonal contraception but unclear how much this is an artefact of baseline differences |
